# A 30-year History of the Emergency Medicine Standardized Letter of Evaluation

**DOI:** 10.5811/westjem.47110

**Published:** 2025-11-26

**Authors:** Jenna S. Hegarty, Cullen B. Hegarty, Jeffrey N. Love, Alexis Pelletier-Bui, Sharon Bord, Michael C. Bond, Samuel M. Keim, Kevin Hamilton, Eric F. Shappell

**Affiliations:** * Rosalind Franklin University of Medicine and Science, Chicago, Illinois; † University of Minnesota Medical School, HealthPartners Institute/Regions Hospital, Department of Emergency Medicine, St. Paul, Minnesota; ‡ Georgetown University School of Medicine, Department of Emergency Medicine, Washington, DC; § Cooper Medical School of Rowan University/ Cooper University Hospital, Department of Emergency Medicine, Camden, New Jersey; || The Johns Hopkins University School of Medicine, Department of Emergency Medicine, Baltimore, Maryland; # University of Maryland School of Medicine, Department of Emergency Medicine, Baltimore, Maryland; ** University of Arizona, Department of Emergency Medicine, Tucson, Arizona; †† University of Maryland Medical System Center for Technology Innovation, Baltimore, Maryland; ‡‡ Massachusetts General Hospital / Harvard Medical School, Department of Emergency Medicine, Boston, Massachusetts

## Abstract

Thirty years ago, education leaders in emergency medicine (EM) developed a standardized letter of recommendation to address limitations of narrative letters of recommendation in the residency selection process. Since then, multiple iterations and improvements with specialty-wide adoption have led to this letter being cited as one of the most essential pieces of a residency application. Based on the experience and success in EM, many other specialties have also now adopted standardized letters of their own. In this paper, we detail the 30-year history of the EM standardized letter including form changes and technological innovations, research and validity evidence, and discussion of research and administrative priorities for the future.

## INTRODUCTION

Emergency medicine (EM) was the first specialty to adopt a standardized letter for residency applications. Noting the shortcomings of narrative letters of recommendation featuring lengthy descriptions and heterogenous content and structure, the Council of Residency Directors in Emergency Medicine (CORD) assembled a task force in 1995 to develop a structured assessment to replace narrative letters that was standardized, concise, and discerning.^1^ The resulting assessment form became the Standardized Letter of Recommendation (SLOR) and debuted in the 1995–1996 EM residency application cycle.

Over the past 30 years, the EM standardized letter has evolved through multiple iterations and advancements including updates to the items and domains assessed, migrating from disseminated paper forms to a centralized electronic database, and development of form variants for subspecialty and off-service rotations. Now known as the Standardized Letter of Evaluation (SLOE),^2^ the EM SLOE has led the way for other specialties that recognized the strength of this approach and developed their own standardized letters of evaluation in subsequent years, such as plastic surgery in 2012, internal medicine and orthopedic surgery in 2017, obstetrics and gynecology in 2022, and more. 3–6 Additionally, the Coalition for Physician Accountability’s Undergraduate Medical Education-Graduate Medical Education Review Committee recommended in 2021 that all specialties develop and move to structured evaluation letters instead of narrative letters.^7^

In this paper, we detail the 30-year history of the EM SLOE including form changes and technological innovations in response to evolving needs and priorities of the broader EM community. This history and accompanying context can inform efforts of those responsible for developing, researching, writing, and interpreting SLOEs by standardizing the language used to describe SLOE versions and variants, summarizing the literature on the topic, and mapping research and administrative agendas for the future.

## STANDARDIZED LETTER VERSIONS

The first version of the EM standardized letter, the SLOR, was produced in 1995 by a task force commissioned by CORD (Table 1).^1^ This letter debuted in the 1995–1996 residency application cycle and featured sections assessing qualifications for EM (commitment, work ethic, ability to formulate a differential and plan, personality) in addition to a global assessment, estimated match list position, and comments (Appendix 1A).From 1995–2011 the SLOR became an influential aspect of the EM residency match process, and the original letter format was iterated upon.^8^

In 2011, CORD re-established the SLOR task force to study, re-evaluate and update the letter.^8^–^10^ The result was the second official version of the letter, the 2012 SLOE (Table, Appendix 1B), which debuted in the 2012–2013 residency application cycle. At this time, the name SLOR was changed to SLOE to better represent that the letter’s purpose was not necessarily to recommend a student, but rather to provide a standardized evaluation of their performance. In addition to the name change came additions and edits to the letter, such as asking which EM rotation this was for the student and the dates during which the student rotated. In Part B, “Qualifications for EM,” the personality question was removed, and questions were added regarding ability to work with a team, ability to communicate a caring nature to patients, and anticipated guidance during residency. The anchors in this section also moved away from adjectives and toward peer comparison. In Part C, “Global Assessment,” Question 2 changed the rank-list options from descriptions of likelihood of matching to numeric anchors (eg, “Top 10%”). Question 2 also added a clarifying question, “Are you on the committee that determines the final rank list?” to understand whether the letter writer had experience with such rankings. Lastly, the narrative section now had a reduced word limit of 250 words or less to encourage letter writers to be more concise, and to decrease the common practice of advertising the institution where the rotation was completed.

Throughout the time spanning these first two iterations of the standardized letter, some authors customized or changed the form.^8^ This variance weakened the SLOE by straying from one of its core tenets: standardization. Many efforts from 2011–2016 were made by the SLOE task force to increase standardization and prevent customization such as providing author guidelines and training (including lectures, workshops, and discussion groups), and advocating for a non-modifiable electronic template.^9^ To further promote standardization in the use of the SLOE, an electronic portal to write and save letters was developed in 2016, in addition to a new letter form referred to as the electronic SLOE (eSLOE) (Table, Appendix 1C).^11^ This change ensured that no alterations could be made to the form, thus standardizing SLOE data for review and comparison. This form also introduced a section after the narrative to describe the institution, both to provide context for the reader and preempt the use of the narrative to describe institutional characteristics. Additionally, the eSLOE website saved all letter information. It produced copies in the correct format, making uploading to the Electronic Residency Application Service system easier for authors, and establishing an electronic database amenable to research and quality improvement initiatives.

To allow authors to provide context for the unprecedented pandemic conditions of the 2020 and 2021 application cycles, the SLOE committee added a single narrative question to the evaluation asking how the student’s rotation was affected by COVID-19. While this change technically makes the 2020 edition of the eSLOE a different version of the standardized letter (Table, Appendix 1D), it is otherwise the same 2016 eSLOE.

Following the addition of the COVID-19 question in 2020, the SLOE committee again re-evaluated and updated the eSLOE resulting in the 2022 electronic SLOE 2.0 (eSLOE 2.0) (Table, Appendix 1E).^12^ The most notable change in the 2022 eSLOE was the addition of criterion-referenced items and removal of some norm-referenced items. This transition was made in step with broader trends in medical education toward assessments that compare performance to a standard as opposed to other trainees. Another competency-based assessment for EM students in use at that time, the National Clinical Assessment Tool for Medical Students in Emergency Medicine (NCAT-EM),^13^ provided helpful context as a field-tested, criterion-referenced clinical assessment to emulate in the 2022 eSLOE.^14^,^15^ A question was added to provide more insight into the sources of information used in compiling the SLOE. Authors could also denote whether this evaluation was based on a rotation taken by all students at the letter writers’ institution or just by EM sub-interns, as each would presumably result in a different grading breakdown. There was also the ability to denote any changes in grading practices to inform comparisons of grades across years. With the transition of US Medical Licensing Exam Step 1 scores to pass/fail and more institutions moving to pass/fail curricula, the SLOE committee added a section regarding test-taking ability, identifying any standardized testing completed during the rotation (eg, National Board of Medical Examiners shelf exam, Society of Academic Emergency Medicine tests, or home- grown assessment).

Given the growing number of SLOE iterations, it is important to standardize the nomenclature to improve clarity in discussions and future literature on this topic. When referring to these evaluations generally and inclusive of SLOR and SLOE versions, we propose reference to the emergency medicine *standardized letter*. When referring to specific versions of the EM standardized letter, we propose referring to the year the version was first used in practice and either SLOR or SLOE, as appropriate (eg, 1995 SLOR, 2016 SLOE). Modifiers to further distinguish versions (eg, 2016 eSLOE or 2022 eSLOE 2.0) may also be used; however, we recommend still including the year in these cases to avoid potential misunderstanding via errors of omission (eg, omitting “2.0” from “eSLOE 2.0” for brevity or by mistake could lead to the reader interpreting this as the 2016 version when the 2022 version was intended, whereas “2022 eSLOE” is unambiguous).

## STANDARDIZED LETTER VARIANTS

From the use of the SLOR through the 2016 SLOE, writers and reviewers began to identify and report to CORD leadership opportunities where clearer differentiation between types of authors and rotations would be beneficial for writers, reviewers, and researchers. These opportunities for clearer differentiation resulted in the creation of multiple SLOE variants. In 2016, the SLOE for Non-Residency-based EM Physicians was introduced (Table 1). This variant removed the item requiring authors to describe where the candidate would reside on their rank list, noting that this question was inappropriate for physicians not involved in a residency program. This new form allowed students to still receive evaluations from this group of authors but provided additional context for reviewers by clearly describing the source of the letter. Additionally, this separation facilitated more granular data for research and quality assurance initiatives. Also released in 2016, the subspecialty SLOE extended evaluation opportunities to include EM subspecialists in toxicology, ultrasound, pediatric EM, and emergency medical services (Table).

The COVID-19 pandemic in 2020 prompted significant restrictions on visiting clerkships nationwide, resulting in limited opportunities for students to receive outside SLOEs. To create more opportunities for students to receive standardized letters in the absence of additional EM clerkship availability, the Off Service Standardized Letter of Evaluation (O-SLOE) was developed (Table 1, Appendix 2G). The O-SLOE expanded access to standardized letters from off-service faculty in non-EM specialties. At this time a question regarding COVID-19 was also added to both the subspecialty SLOE and the SLOE for non-academic emergency physicians (Table 1, Appendix 2A, 2D).

All three variants of the SLOE were updated by the CORD SLOE Committee again in 2022 to match the updated 2022 SLOE (Table, Appendix 2B, 2E, 2H). The variants were also added to the eSLOE database at that time. The latest addition to SLOE variants in 2024 was a bar at the top of each PDF with a unique color to signify each variant, making it clear to SLOE readers which type of SLOE variant they were reading (Table, Appendix 2C, 2F, 2I).

## RESEARCH

Highlights of SLOE research from author experience and PubMed search for “standardized letter of evaluation” include a broad scope of topics. Past research has highlighted the SLOE’s value as one of the most heavily weighted aspects of an applicant’s file.^9^,^13^,^16^ When compared to narrative letters of recommendation, the EM SLOE was interpreted faster by recruitment committees and had higher interrater reliability.^17^

Research investigating the process of how SLOEs are written has noted an increasing proportion of SLOEs authored by groups compared to those authored by individuals.^18^ Program directors in EM have cited increased trust of group SLOEs compared to those authored by individuals despite limitations noted in past analysis of group SLOE-authorship processes.^8^,^9^,^19^ This may be due to slight but statistically significantly higher ratings seen in individual SLOEs compared to group SLOEs, which some may interpret as grade inflation in individual SLOEs. It is worth noting, however, that these score differences are smaller and even reversed when comparing only individual SLOEs written by clerkship directors to group SLOEs, suggesting that clerkship directors authoring individual SLOEs exhibit little to no grade inflation compared to group SLOE authors.^18^ Data presented at the 2025 CORD Academic Assembly also has linked the quantity of SLOEs authored per year with rating trends, noting that lower volume SLOE author(s) gave higher mean ratings compared to high-volume author(s) for both individual and group SLOEs.^20^

Trends in ratings by writer experience and home vs away rotations have also been explored, noting higher ratings in less experienced writers and home rotations.^21^,^22^ While it is encouraging that high-volume author(s) and clerkship director ratings are similar to group SLOE ratings on average, optimizing standardization of ratings across all author types remains a potential growth area for the SLOE. Form updates and consistent messaging and training efforts through CORD have been shown to decrease evidence of rating leniency,^11^ as has defaulting score selections to the midpoint of the range and creating a pop-up notification for when score extremes are selected^23^; however, recent evidence suggests persistence of variable rating practices across institutions that warrants continued efforts in this area.^24^

The competitiveness of applicants based on SLOE information has also been explored through the lenses of simultaneous goals of (1) optimizing match outcomes for applicants and (2) providing programs with stratifying performance information. Analysis of match outcomes for applicants with lower ratings in one study shows increased risk of not matching, but lower ratings did not preempt a successful match.^25^ Another study noted that adherence to rating standards did not seem likely to increase risk of applicants failing to match in EM.^26^ Both of these studies support the notion that whole rating scales can and should be used, although with consistency and transparency to decrease the risk that authors see lower rankings as outlier red flags, which has been described in a qualitative study investigating how SLOEs are interpreted.^19^

Multiple recent studies demonstrate a high degree of faculty consensus regarding the level of competitiveness of an applicant based on the SLOE.^13^,^27^,^28^ These studies also show promise for algorithms to predict consensus levels of competitiveness. These models outperformed artificial intelligence software when comparing their ability to predict faculty consensus rankings of competitiveness.^29^ How these algorithms can be operationalized to improve the application process is an ongoing area of discussion, but this could involve applicant-facing applications such as broad competitiveness feedback to tailor application quantity and breadth, or program-facing applications such as competitiveness estimations to which faculty ratings could be compared to assess for potential bias or to cut down on time needed for reviews.

There have been limited investigations into the association of SLOE ratings with future performance.^30^–^32^ Published studies face challenges of small sample sizes and use of unvalidated outcome measures in two studies. In the study assessing the association between SLOE and Accreditation Council for Graduate Medical Education (ACGME) Milestones ratings, only one year of Milestones data was used, which limits the scope of these results.^31^ National data presented at the 2025 ACGME conference, however, shows a clear association between algorithm-derived SLOE competitiveness and multiple measures of residency Milestones performance including mean first and last Milestone ratings by competency and the binary outcome of residency completion.^33^ Future SLOE research should continue to prioritize studies linking SLOE ratings to future performance.

While many strengths of the EM standardized evaluation have been discovered, areas for improvement have also been identified. Literature suggests that both sex-based and racial bias are demonstrated in certain components of the eSLOE.^34^–^36^ There is also evidence that institutional rating patterns and adherence to written standards vary widely, which has raised long-standing concerns about grade inflation and its impact on the ability to stratify applicant performance.^8^,^11^,^24^,^26^,^37^,^38^ Additionally, a review of validity evidence for the 2016 SLOE highlights areas of improvement to consider, although more recent research has addressed some of these concerns.^39^

## NEXT STEPS

Emergency medicine has led the field in standardized letters for the residency application process for the past 30 years. Looking forward to how EM can lead in the next 30 years, several areas stand out. These areas include mitigating the influence of bias on standardized letter of evaluation assessments, continuing to adapt the SLOE instructions, questions, data points, and form to improve response processes and data quality (including efforts to curb, or at least track and facilitate adjustment for, grade inflation), and further bolstering the validity evidence for the SLOE through research including measuring the association of SLOE ratings with future performance.

## Supplementary Information


