# Supplementary material for: A 30-year History of the Emergency Medicine Standardized Letter of Evaluation
Source: West J Emerg Med. 2025 Nov 26;26(6):1544–8. doi: 10.5811/westjem.47110 (PMC12698172; doi:10.5811/westjem.47110)
Supplement: Supplementary file 1 [file wjem-26-1544-s001.docx]

**Appendix 1A-E. Emergency Medicine Standardized Letter Versions**

1. 1995 SLOR
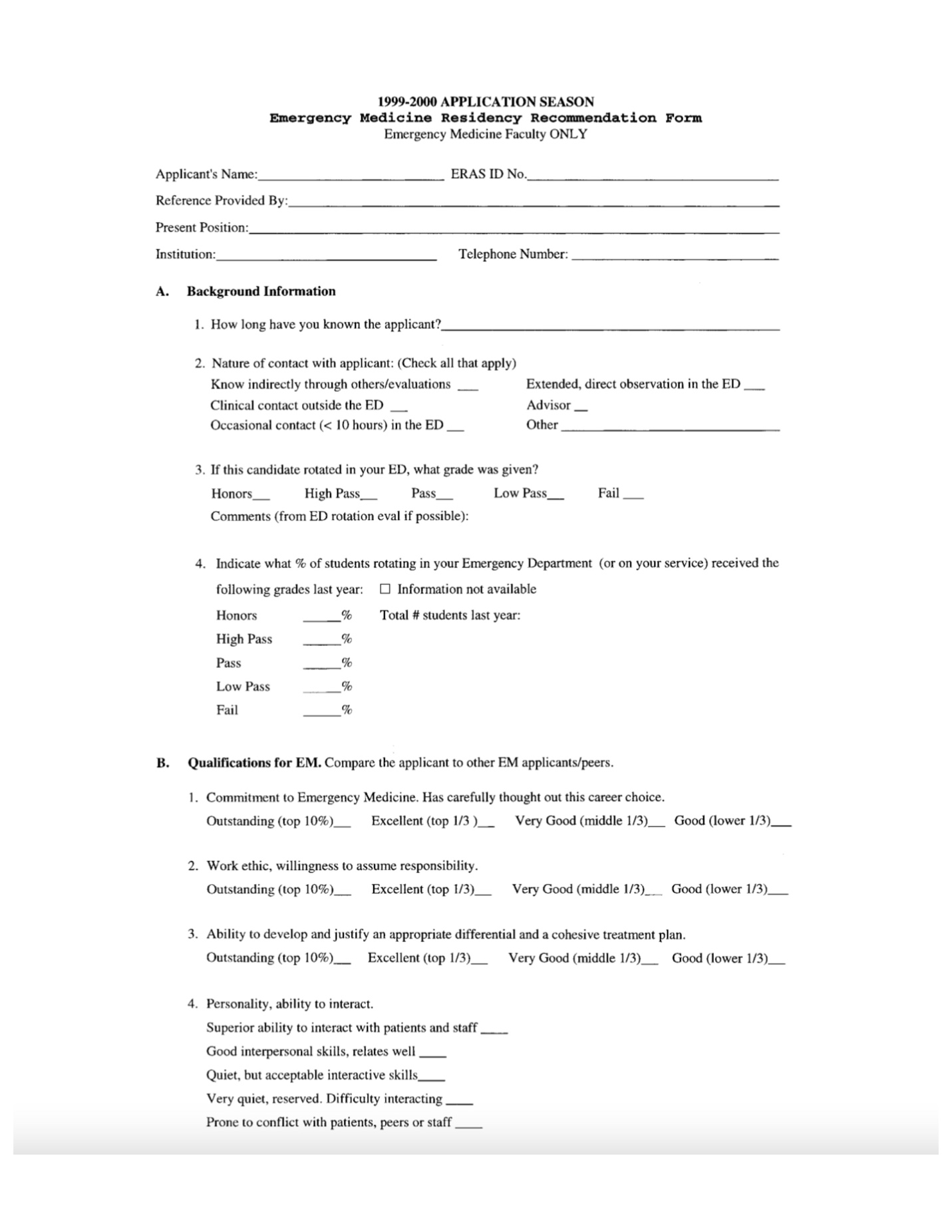

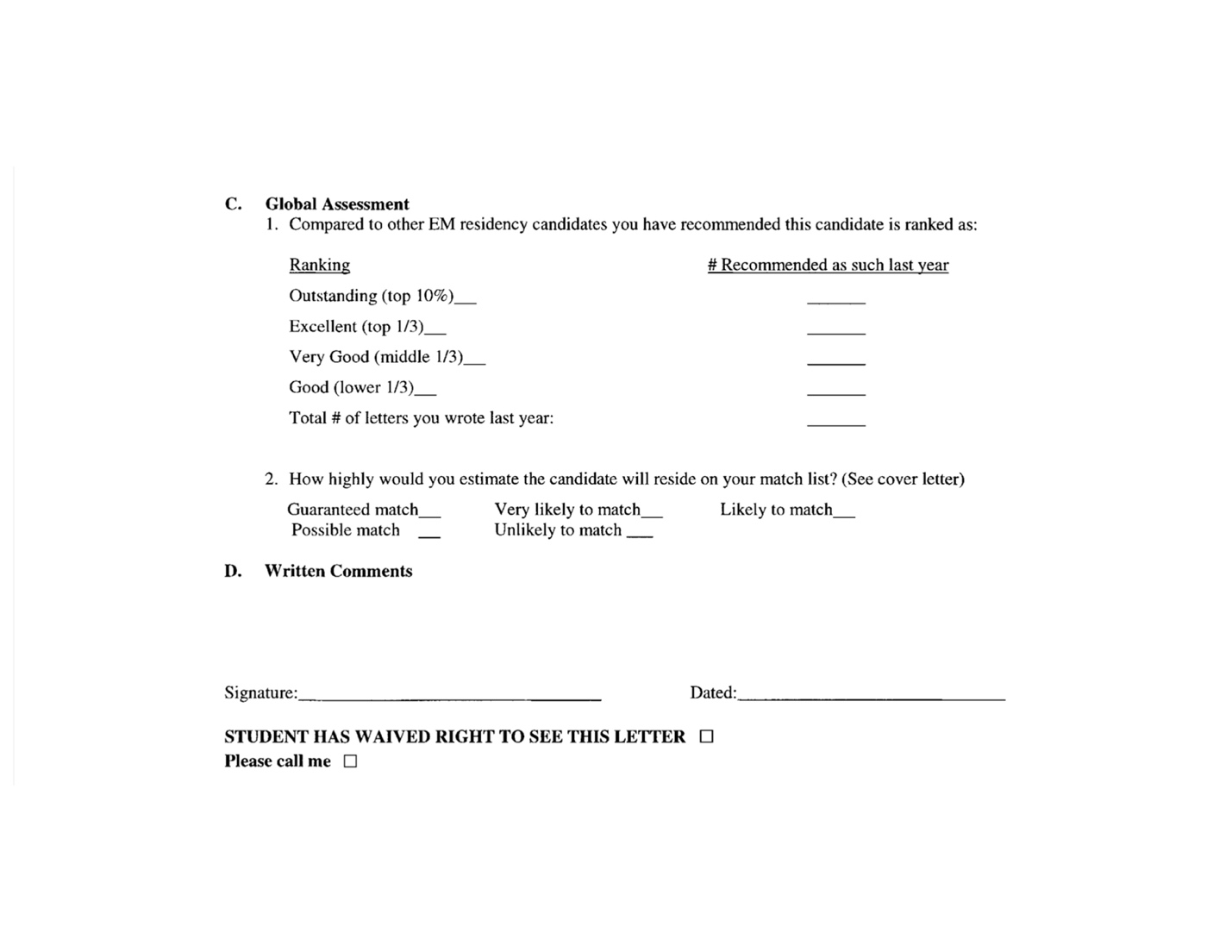

2. 2012 SLOE
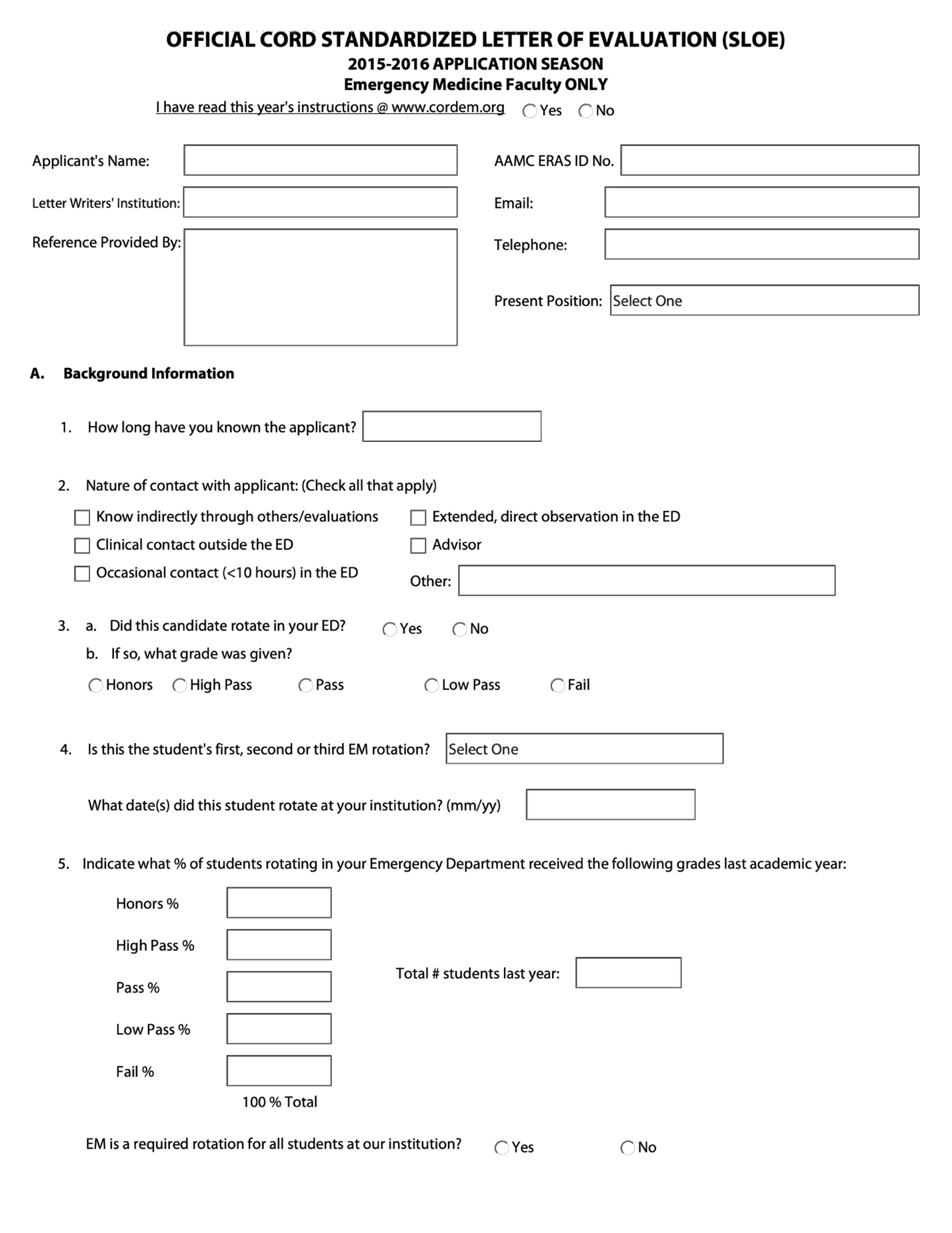

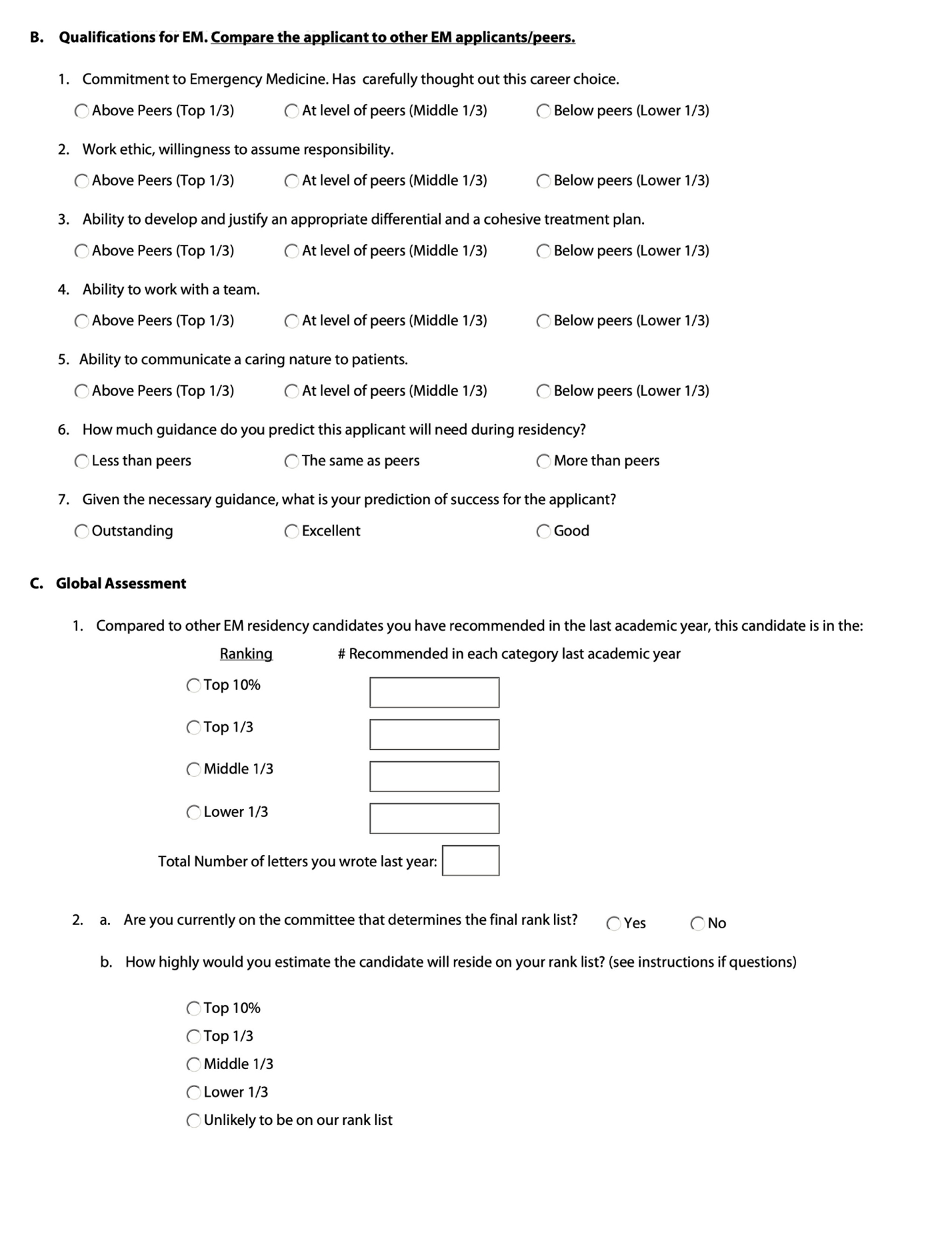

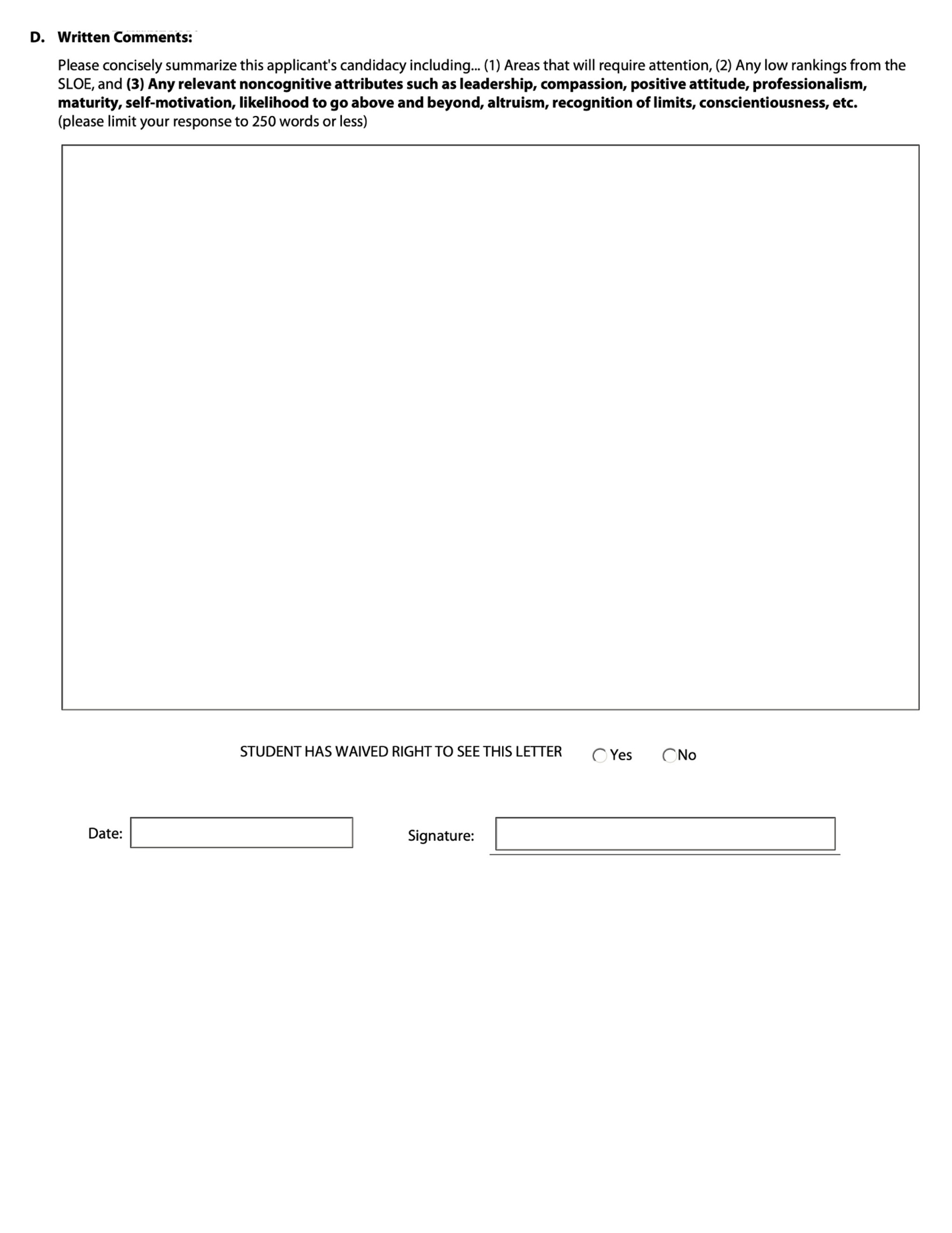

3. 2016 SLOE
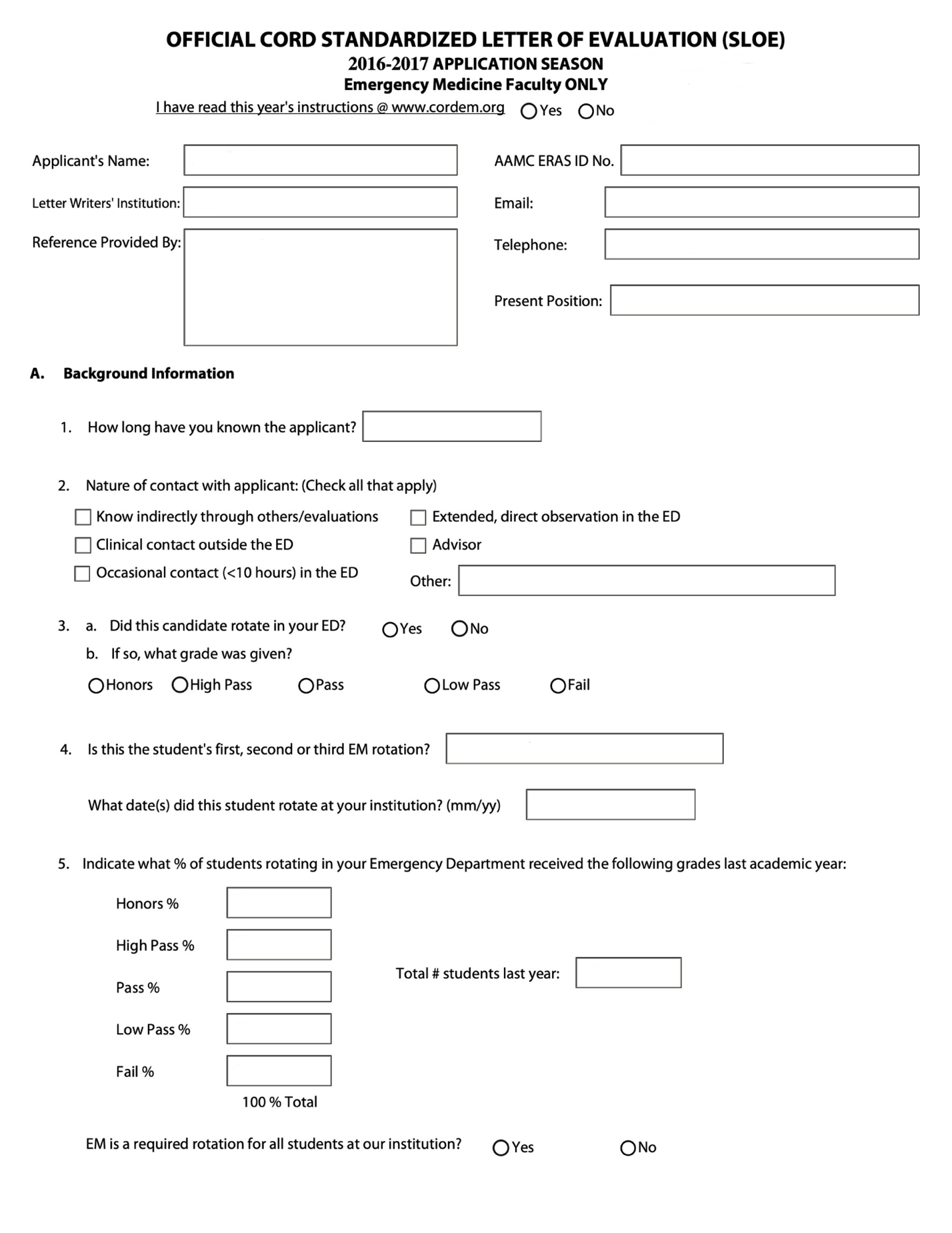

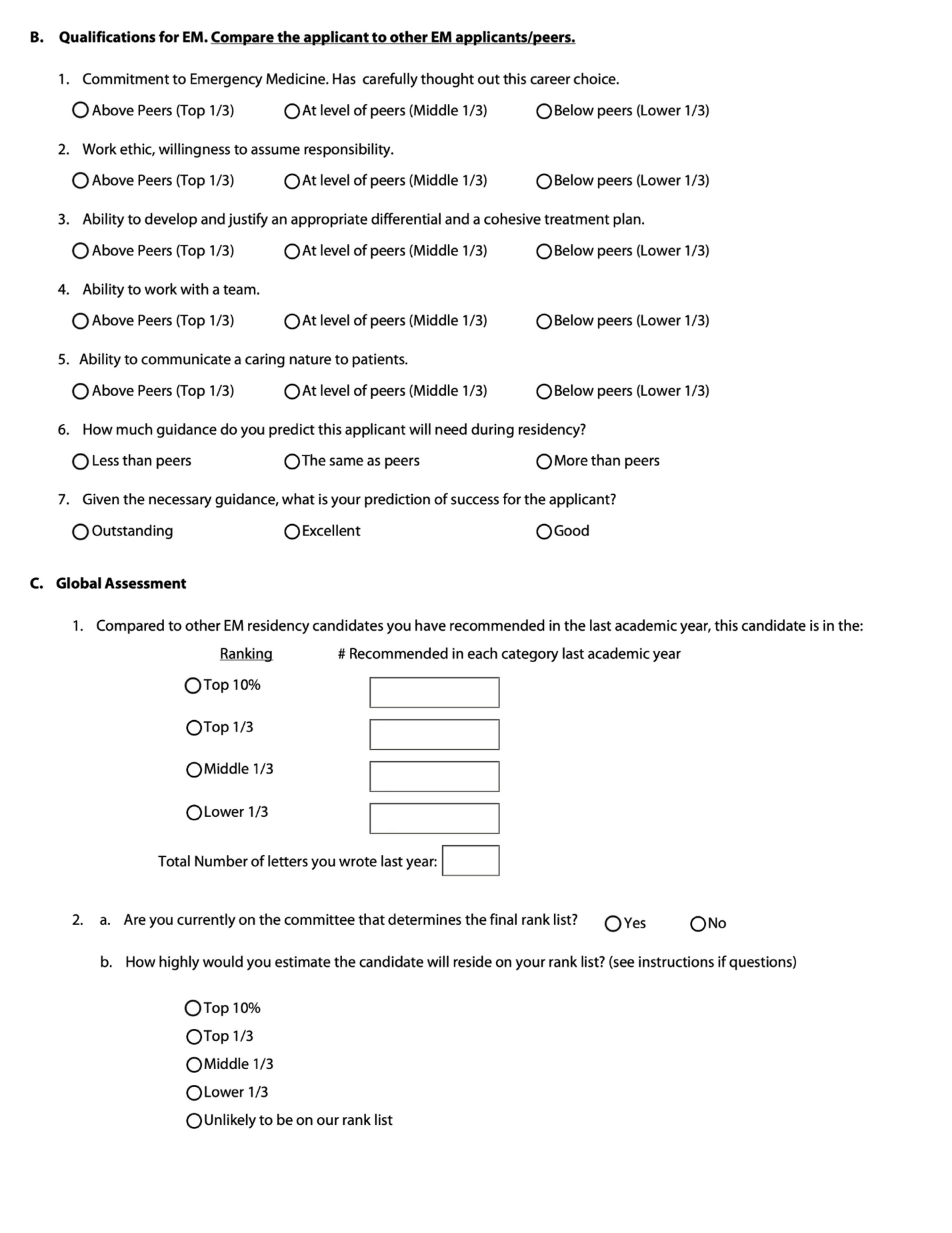

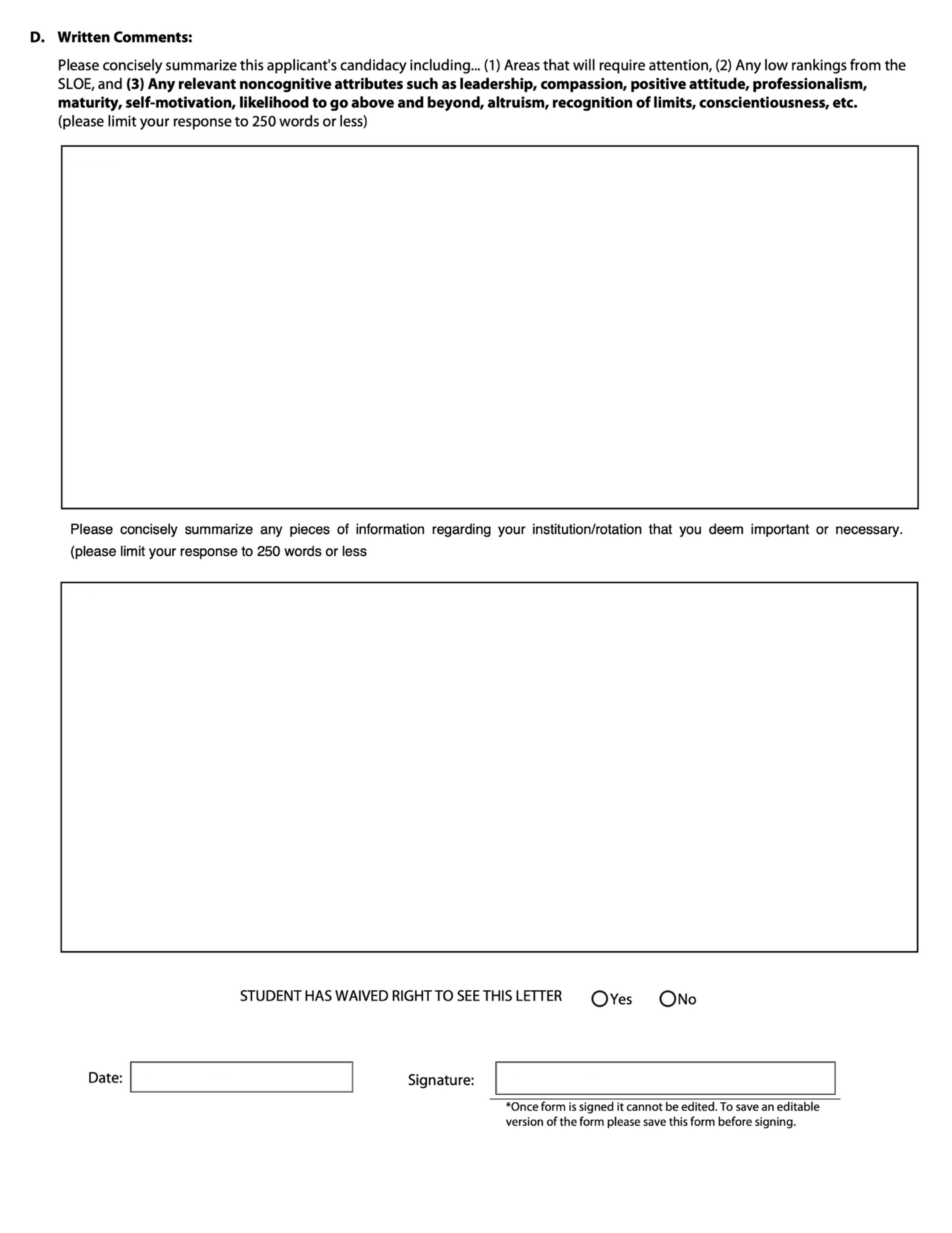

4. 2020 SLOE
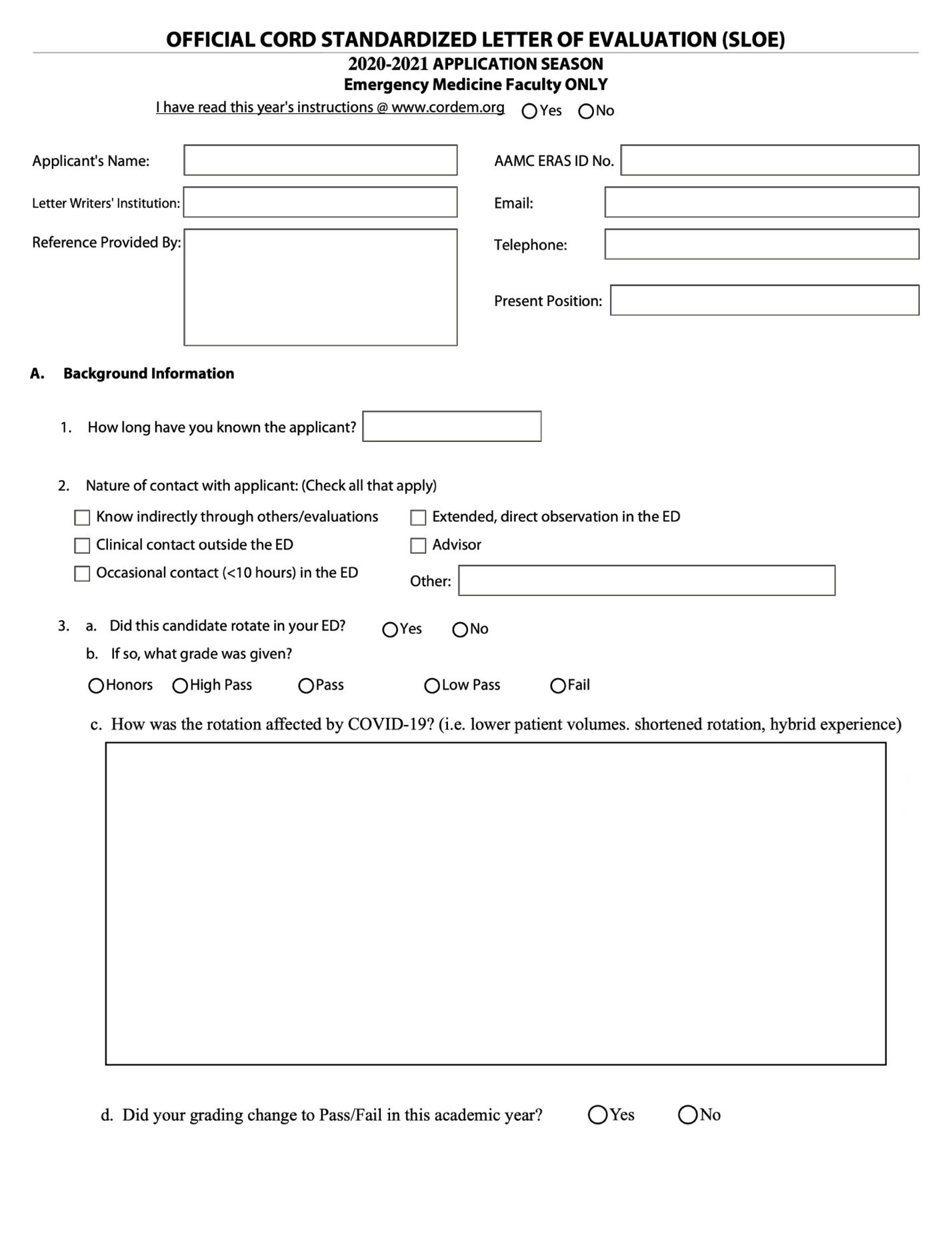

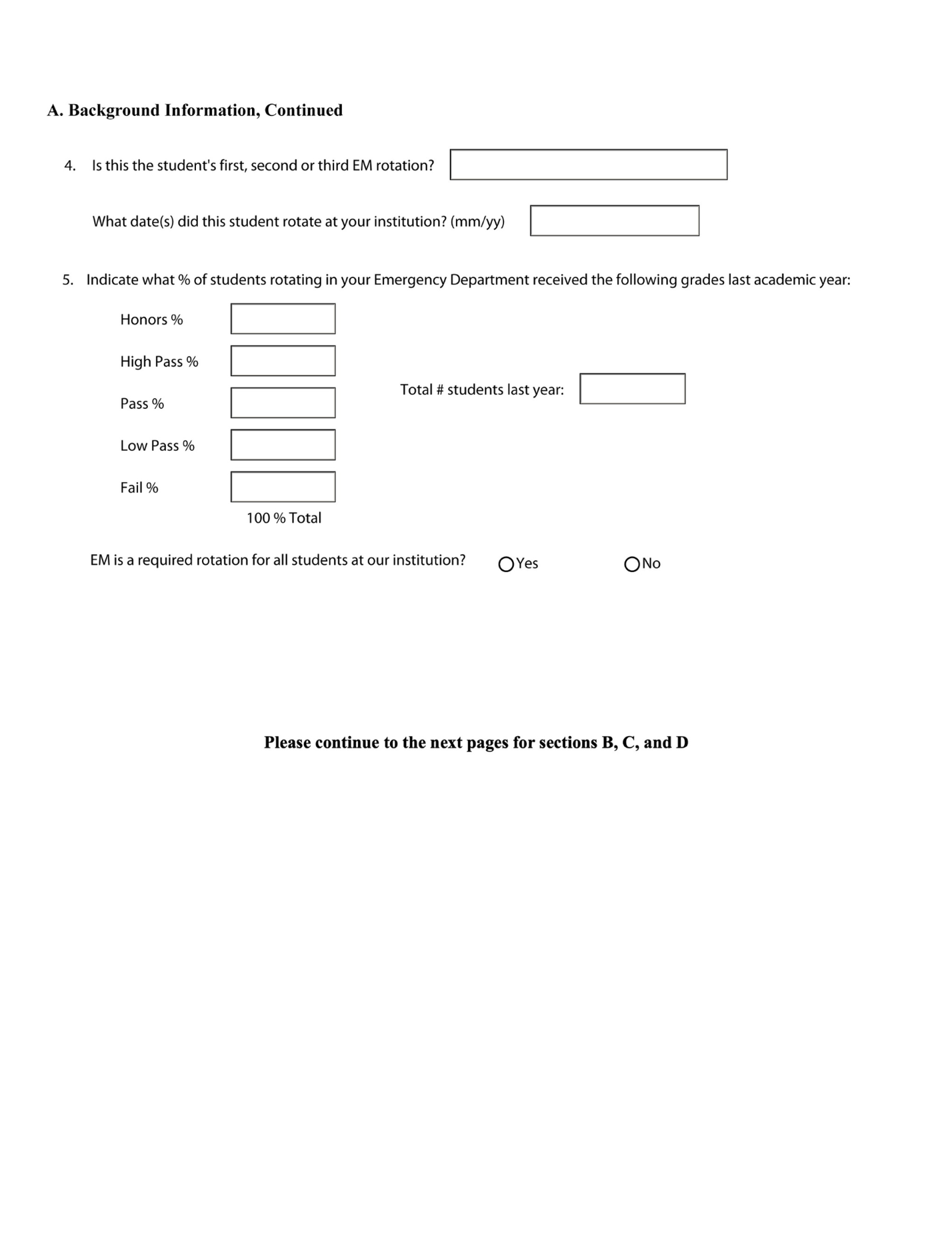

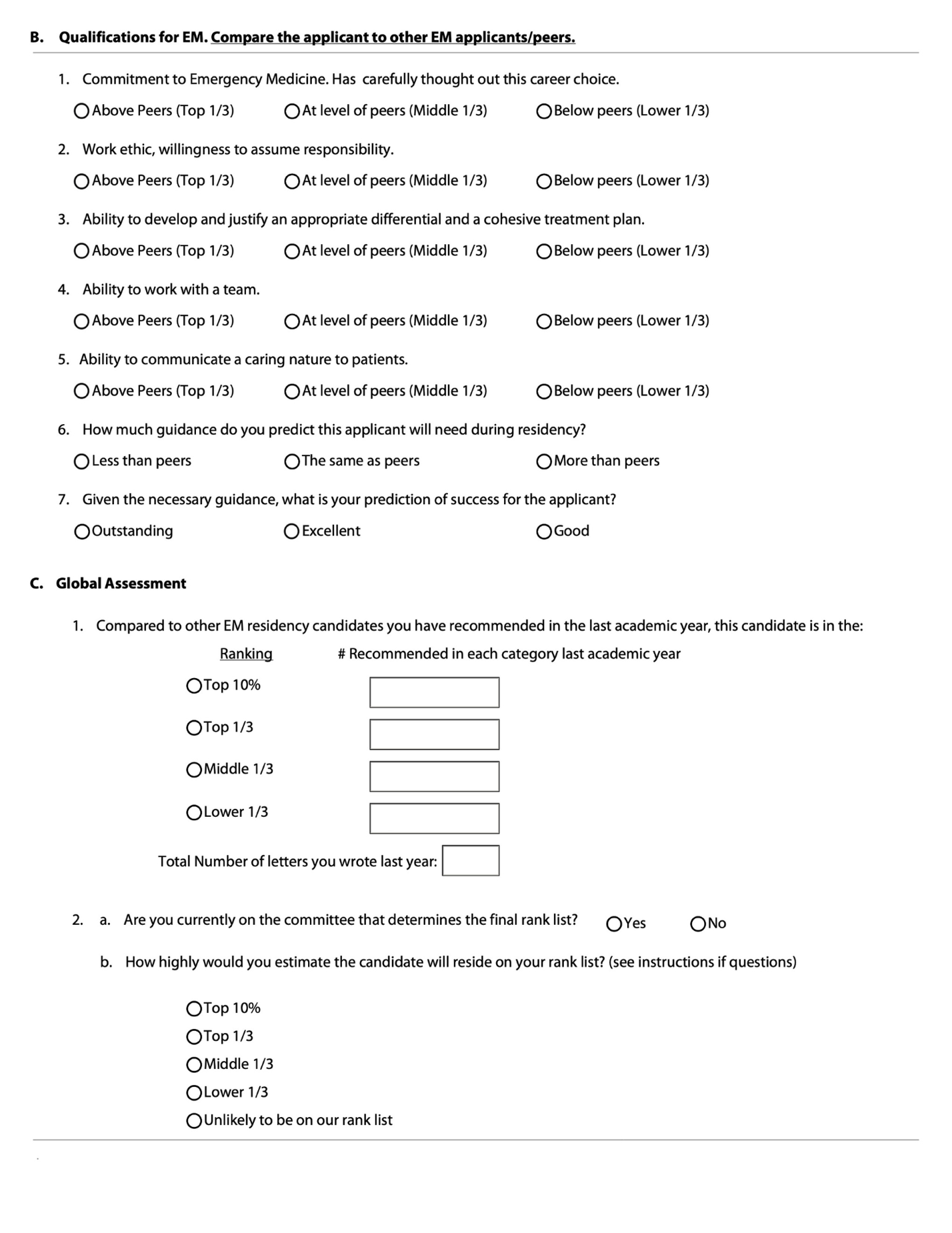

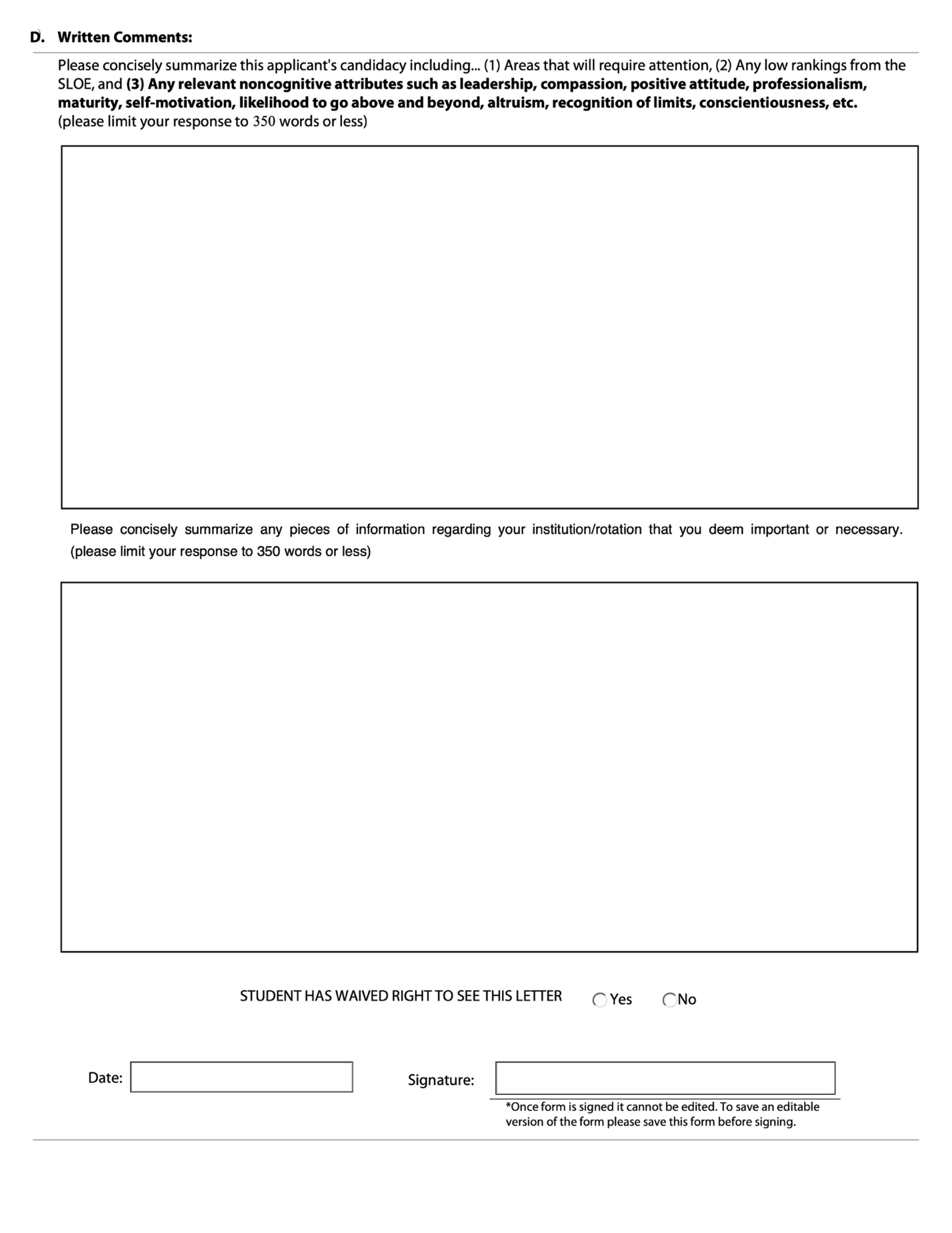

5. 2022 SLOE
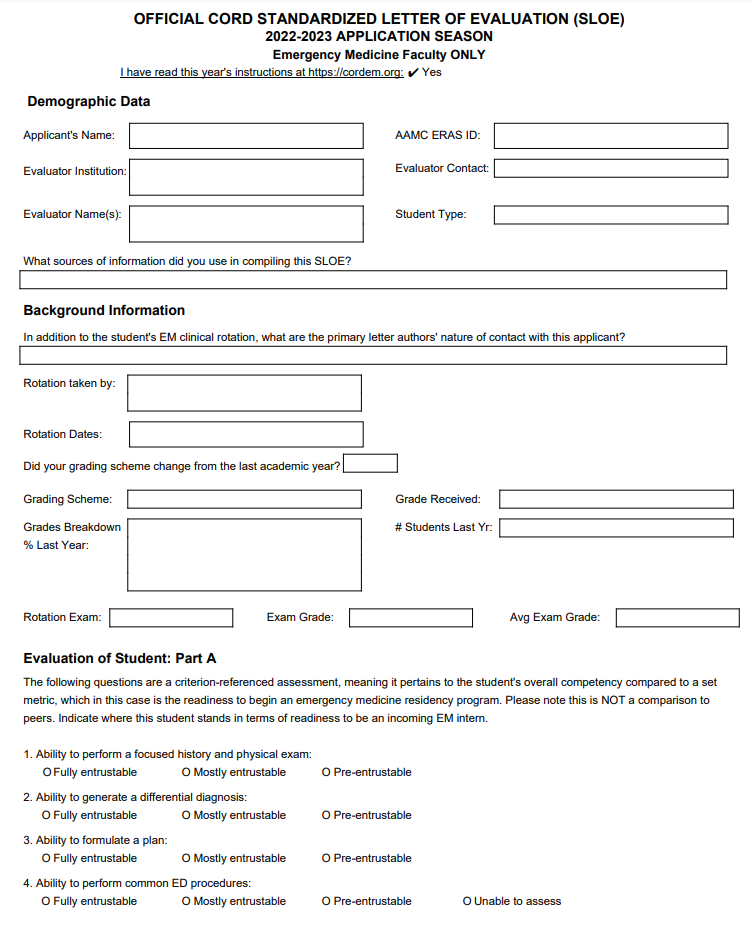

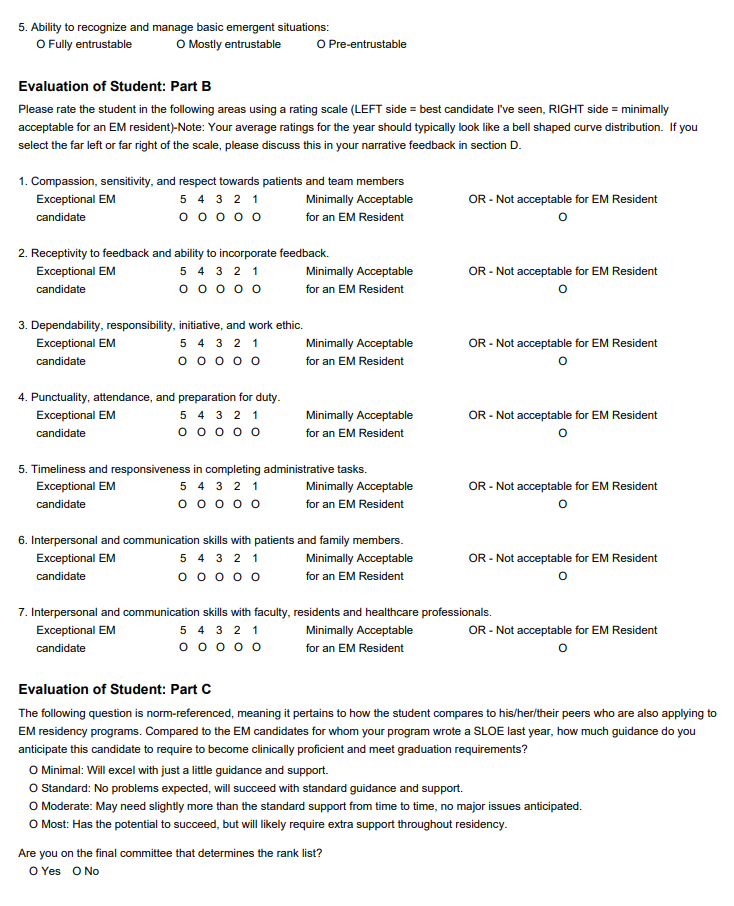

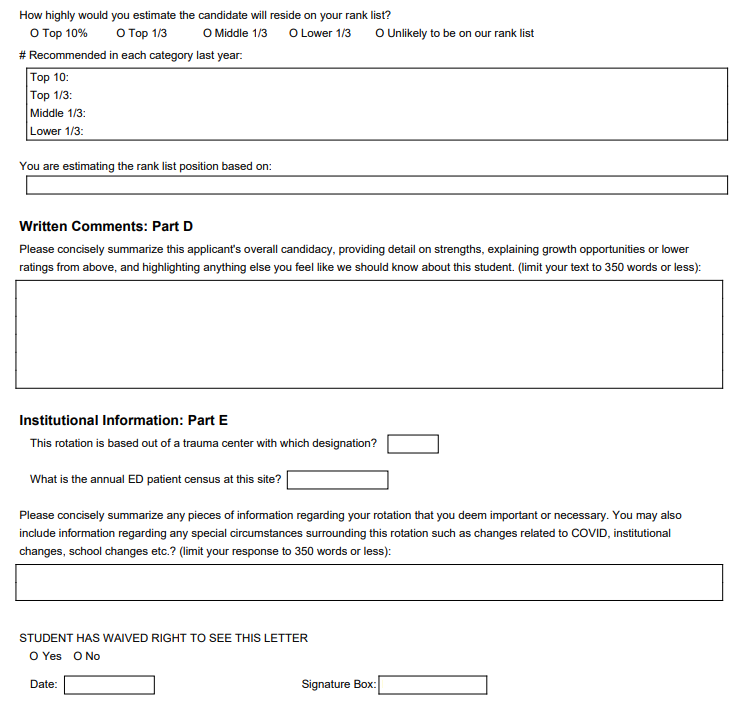


**Appendix 2A-I. Emergency Medicine Standardized Letter Variants**

1. 2020 SLOE for Non-academic Emergency Physicians
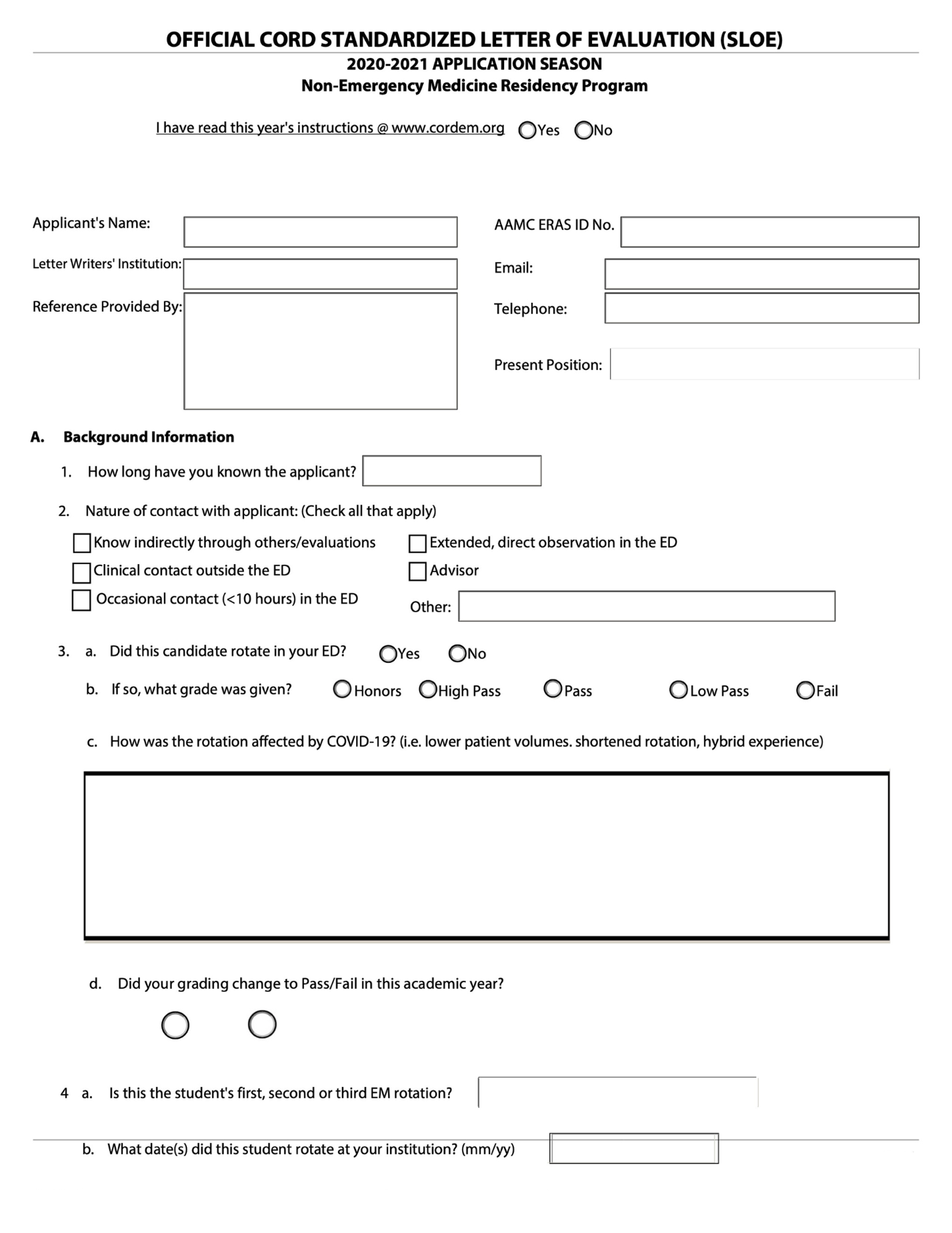

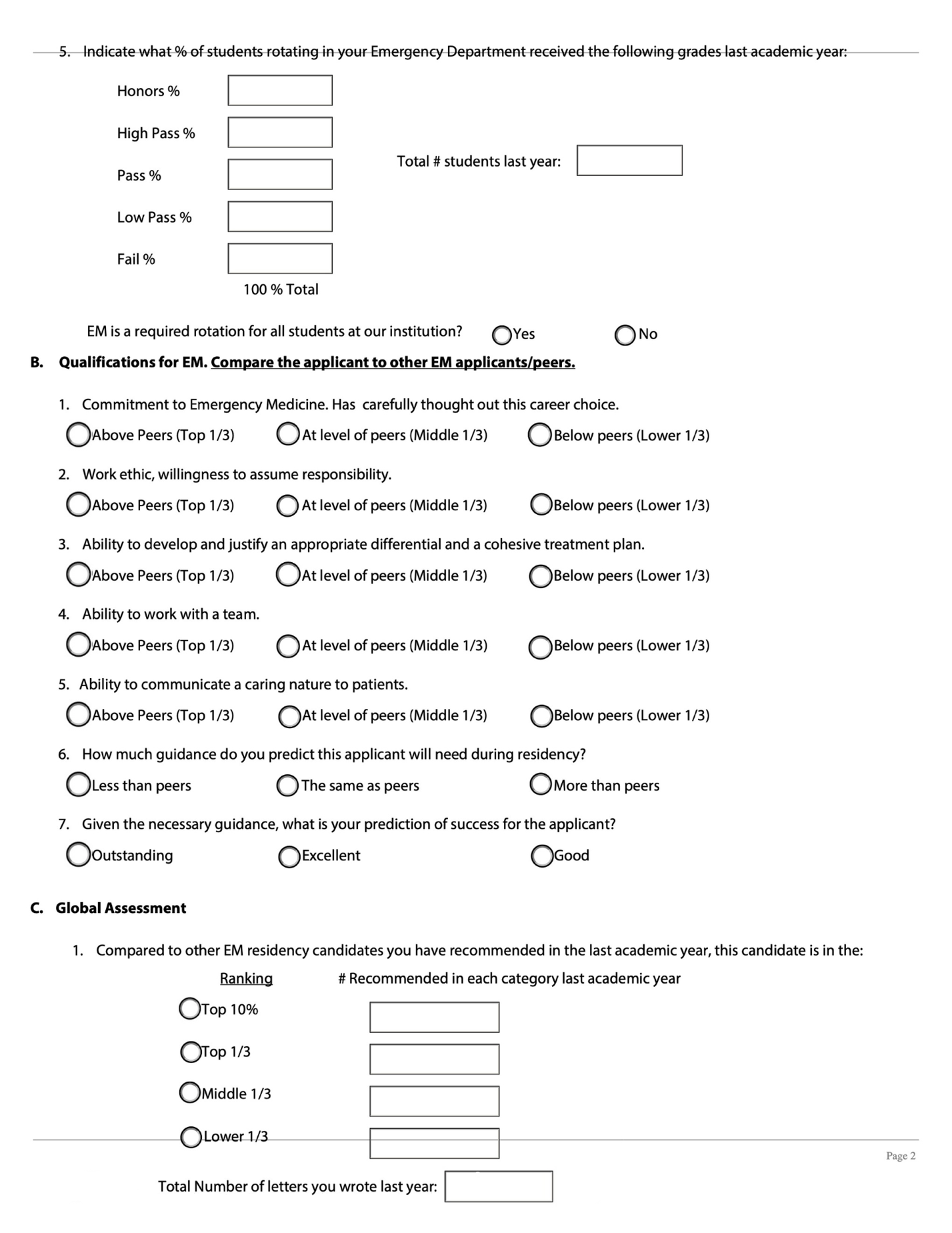

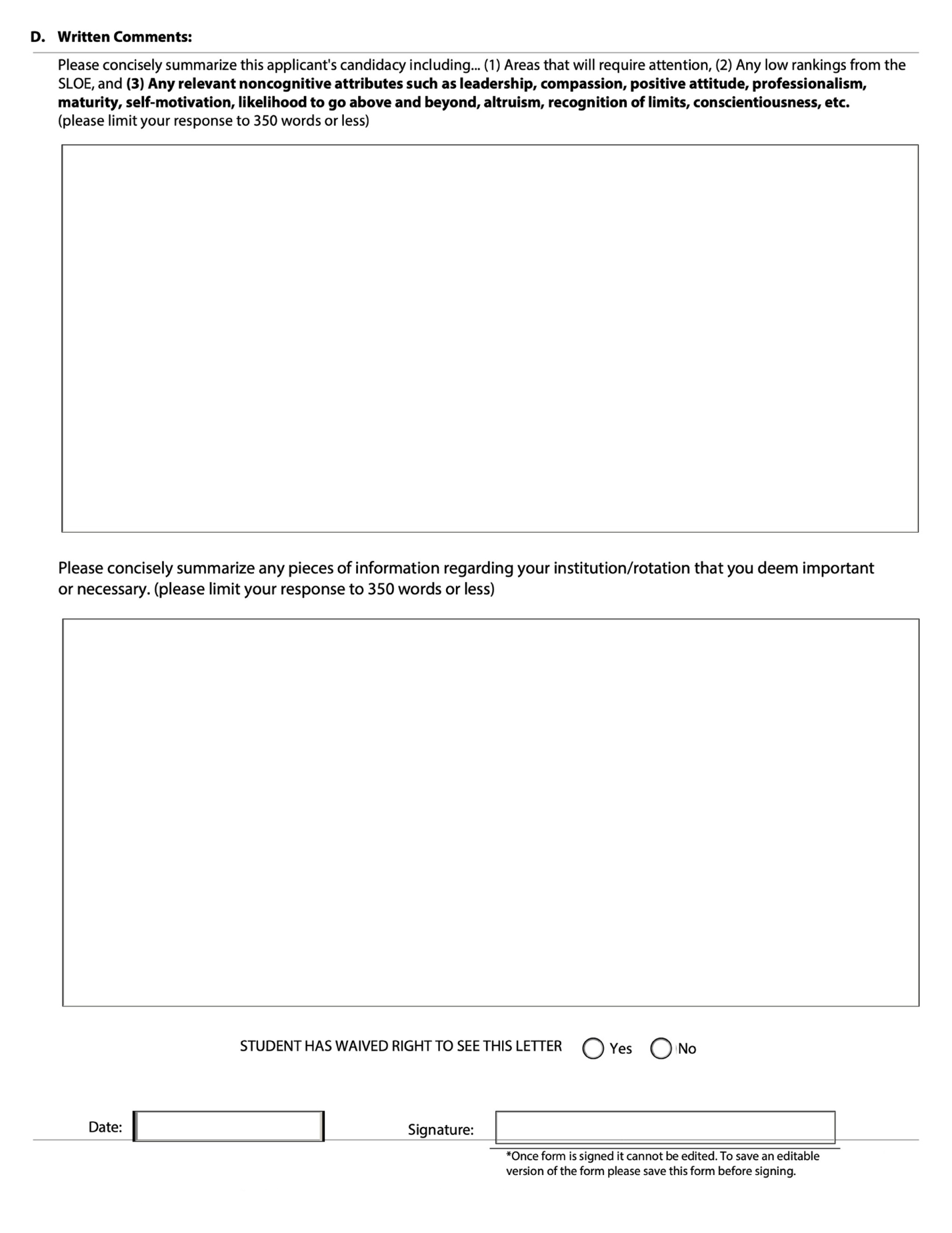

2. 2022 SLOE for Non-academic Emergency Physicians
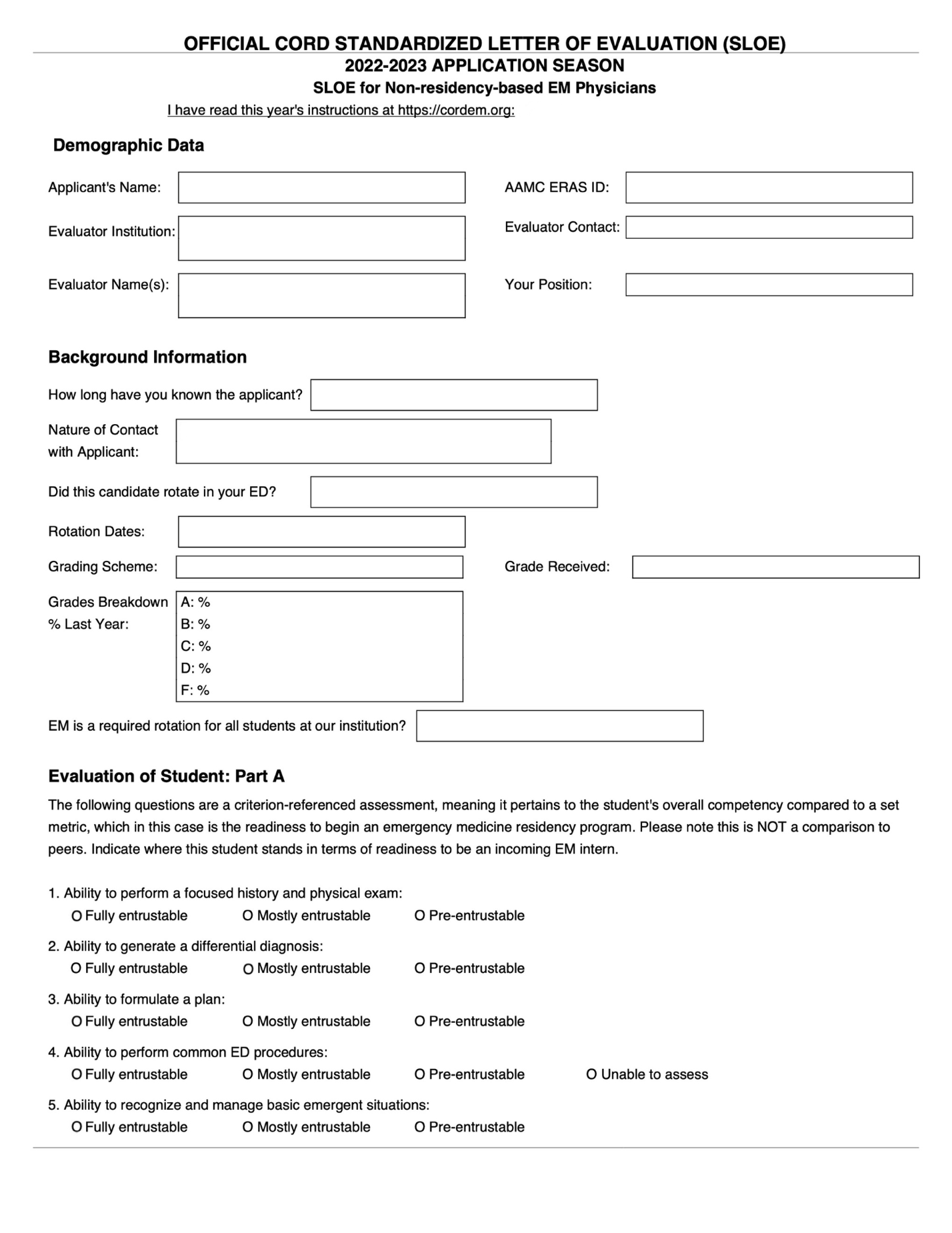

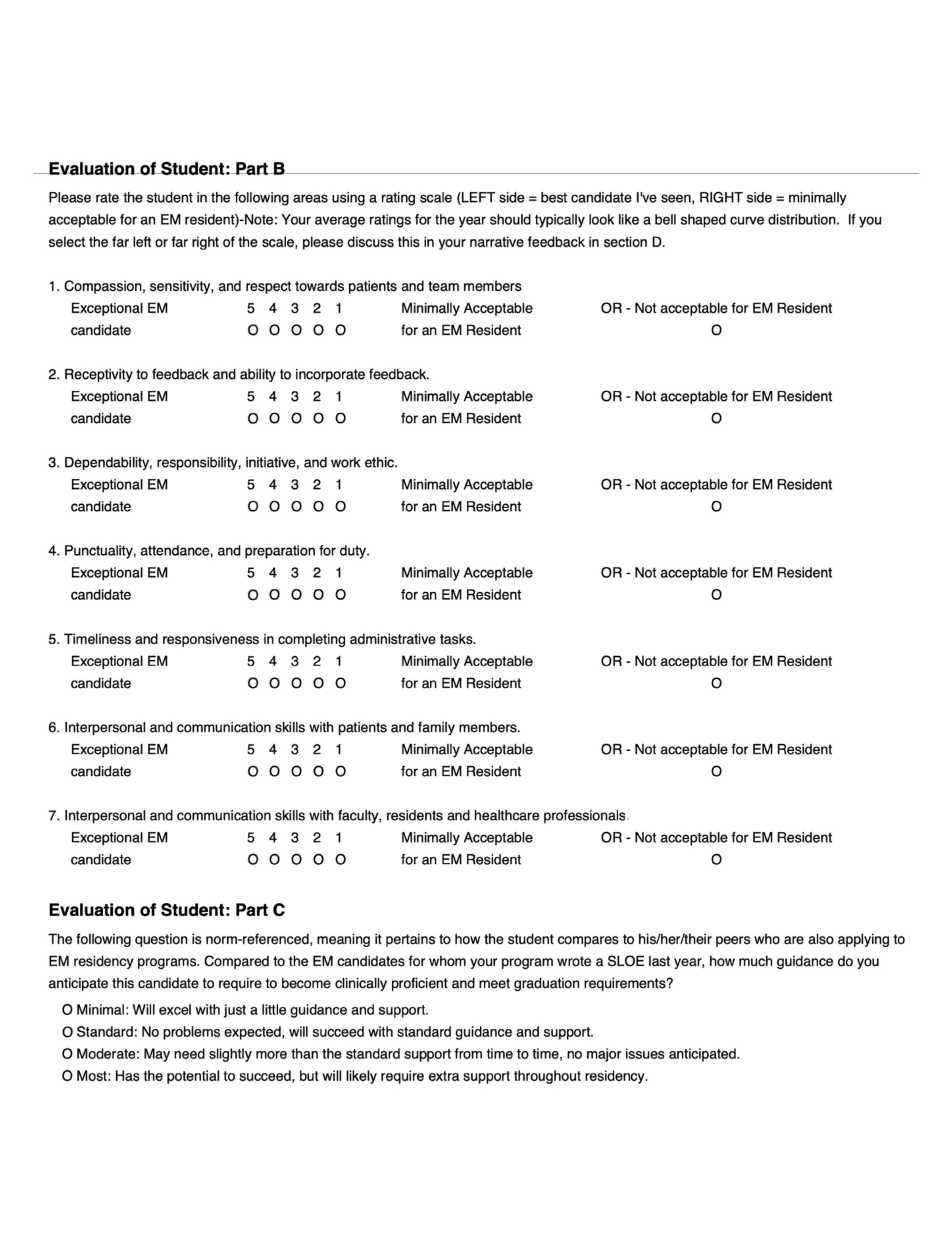

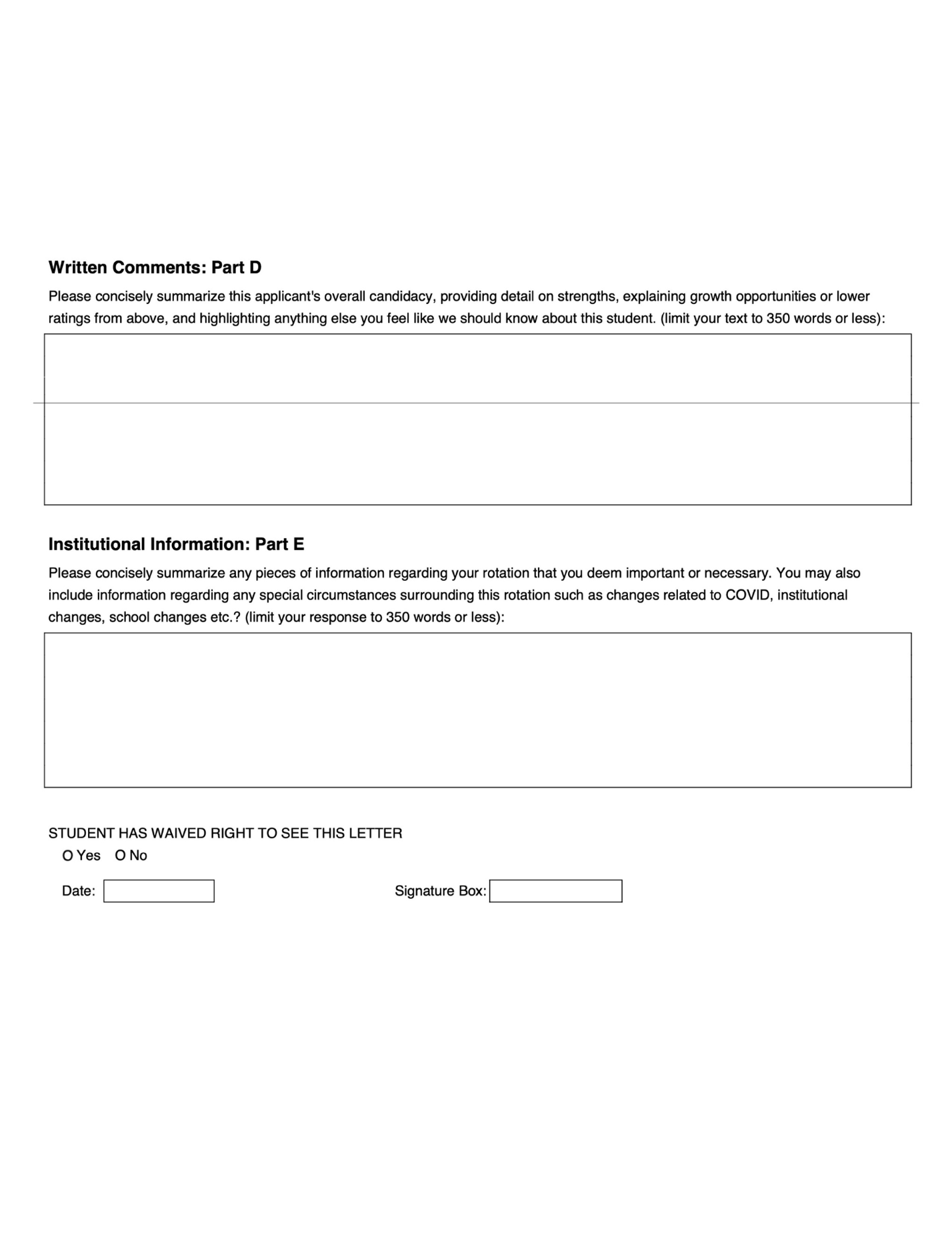

3. 2024 SLOE for Non-academic Emergency Physicians
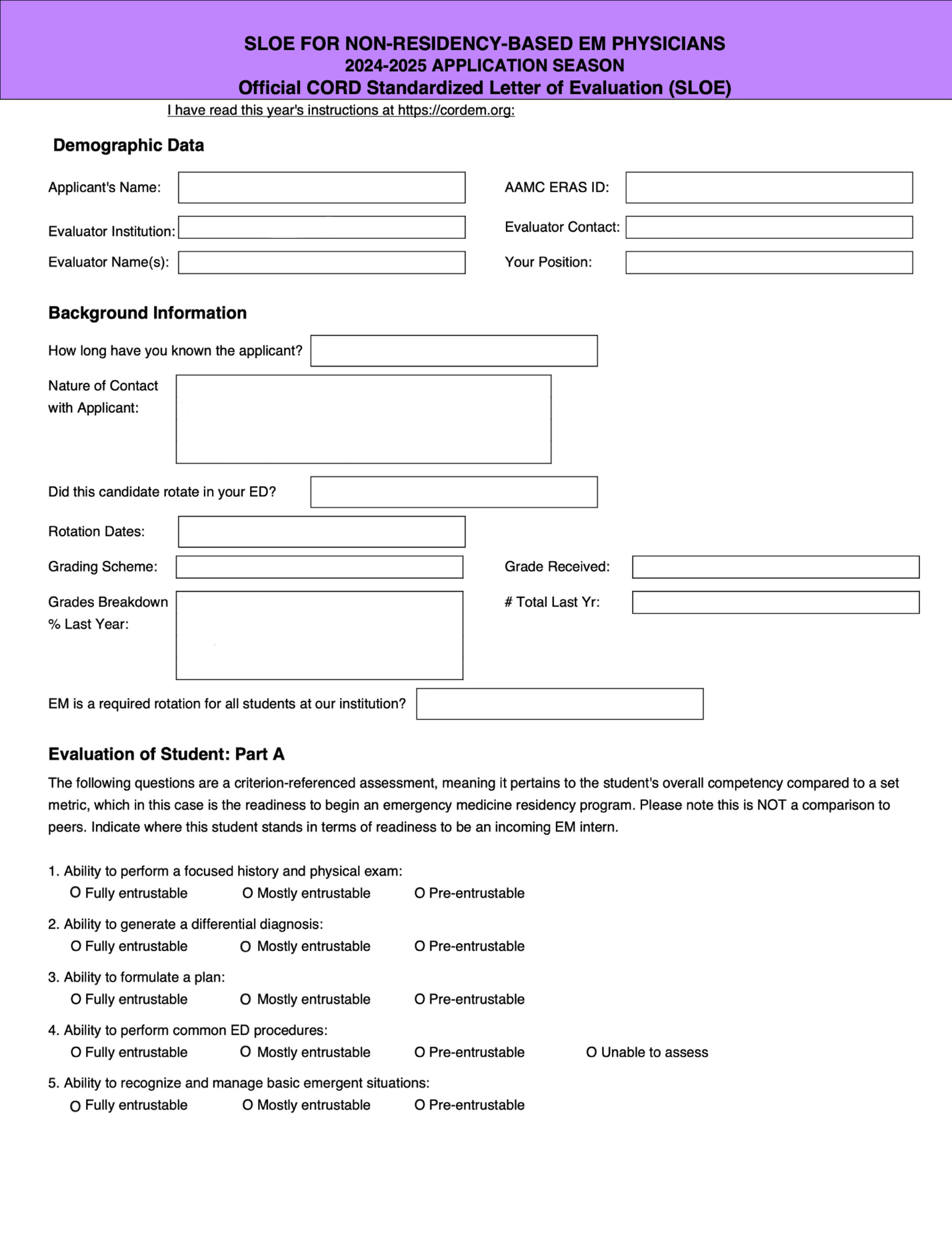

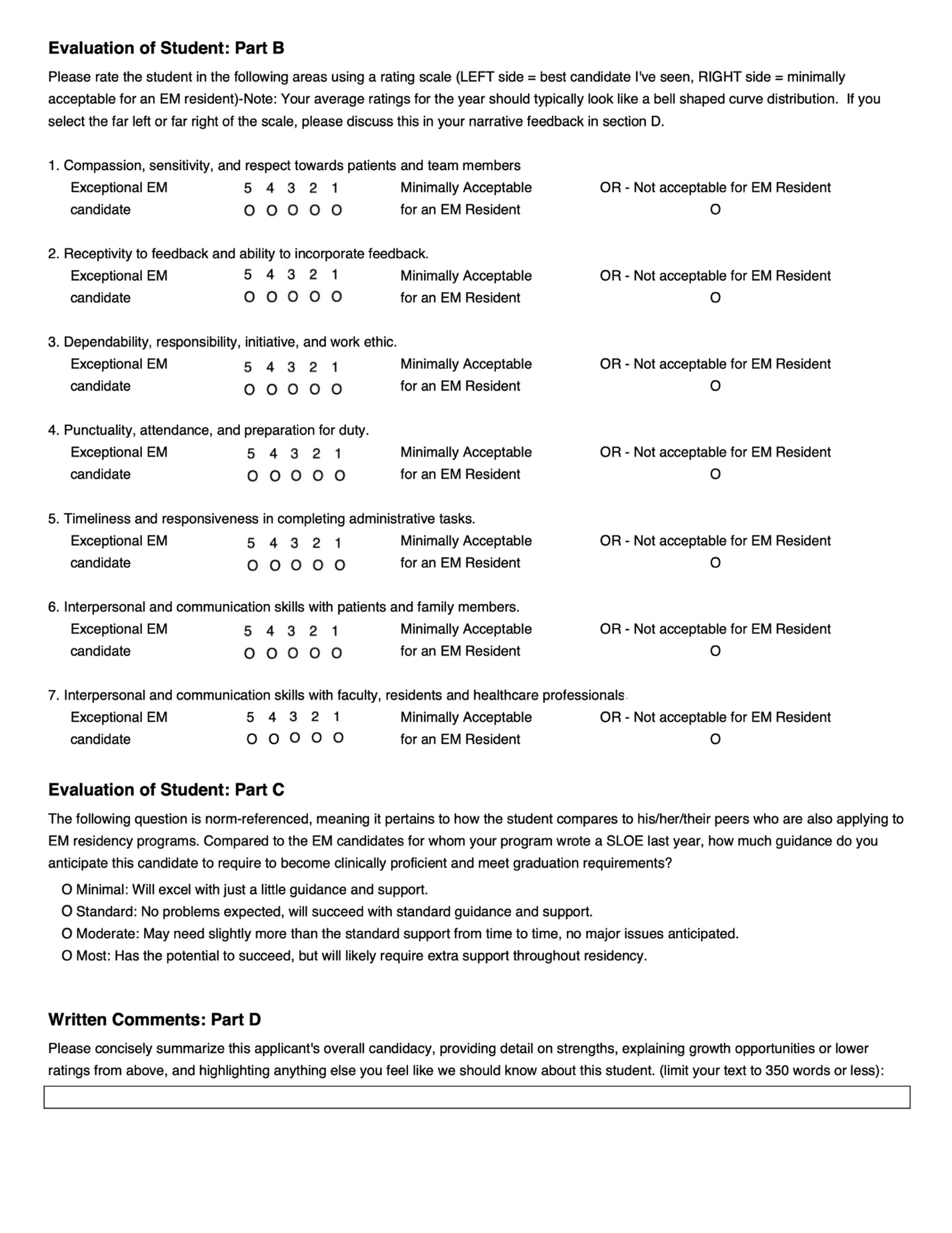

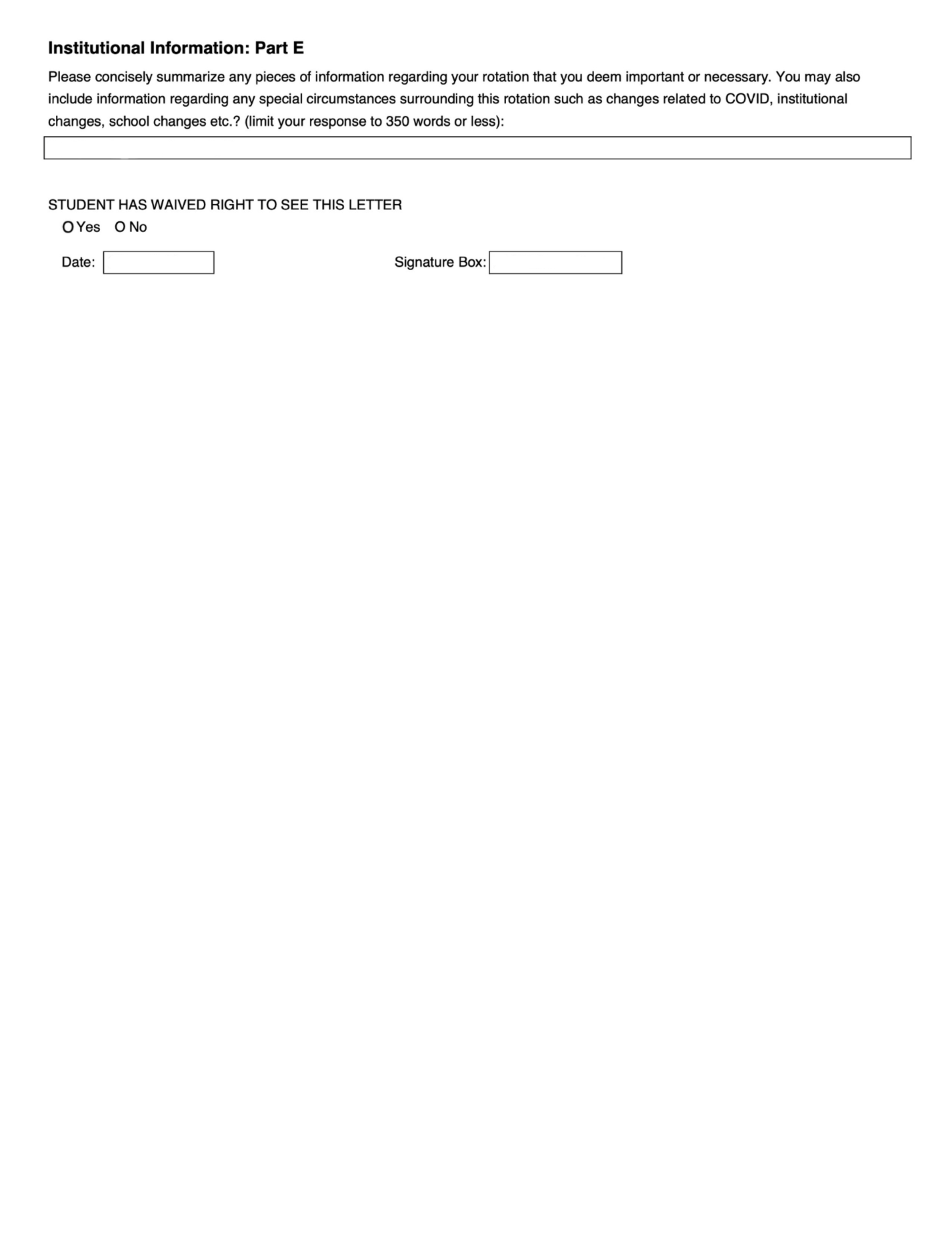

4. 2020 Subspecialty SLOE
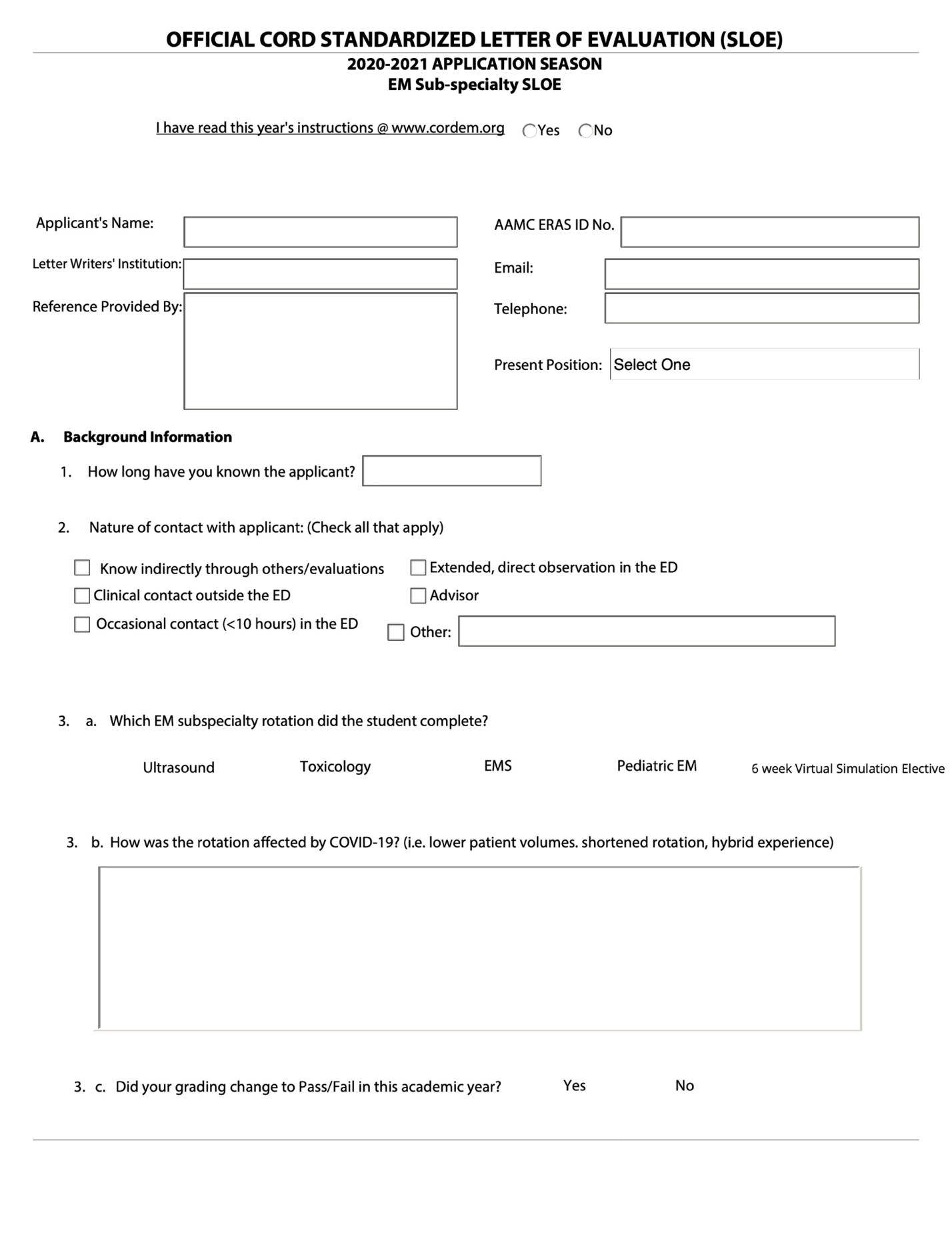

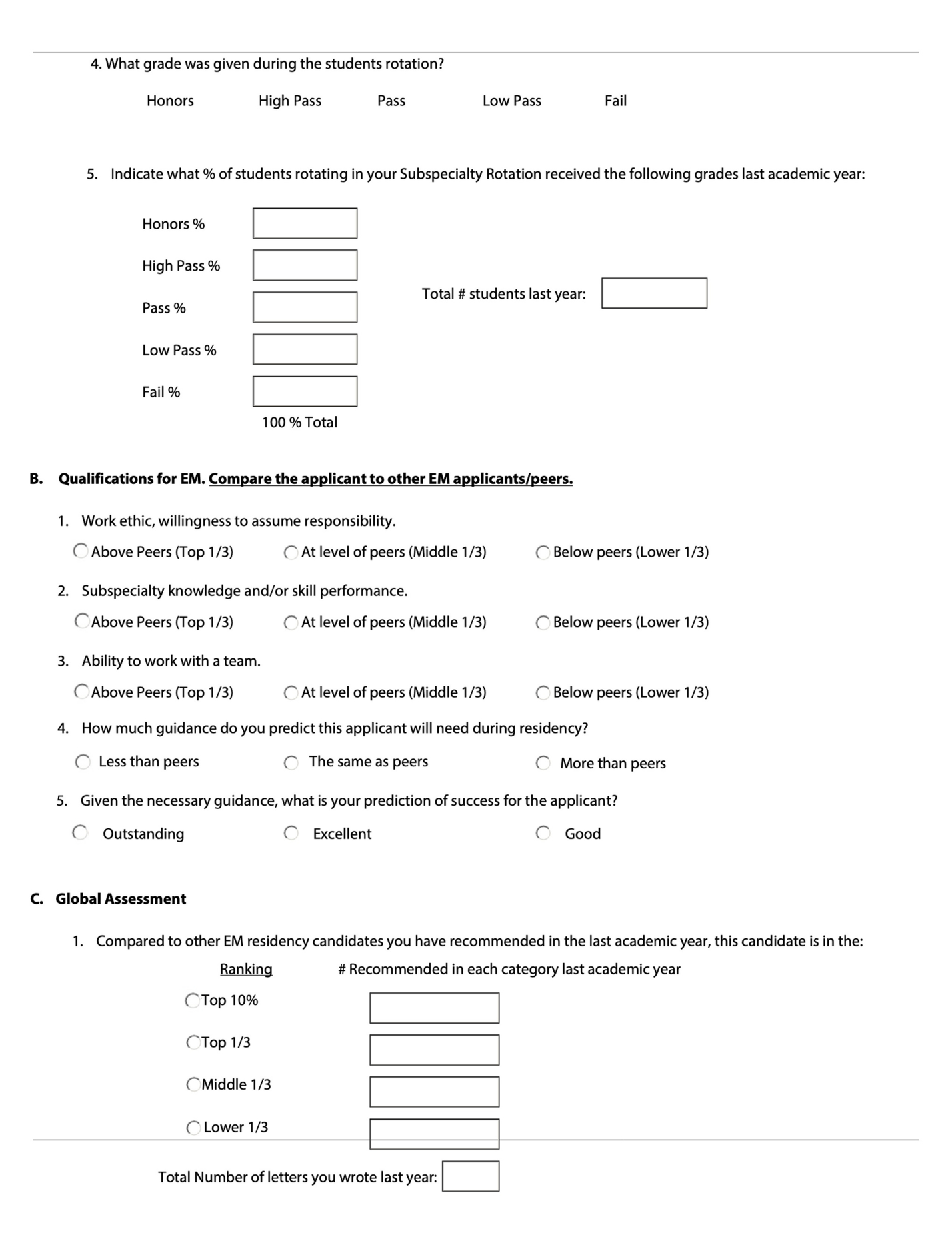

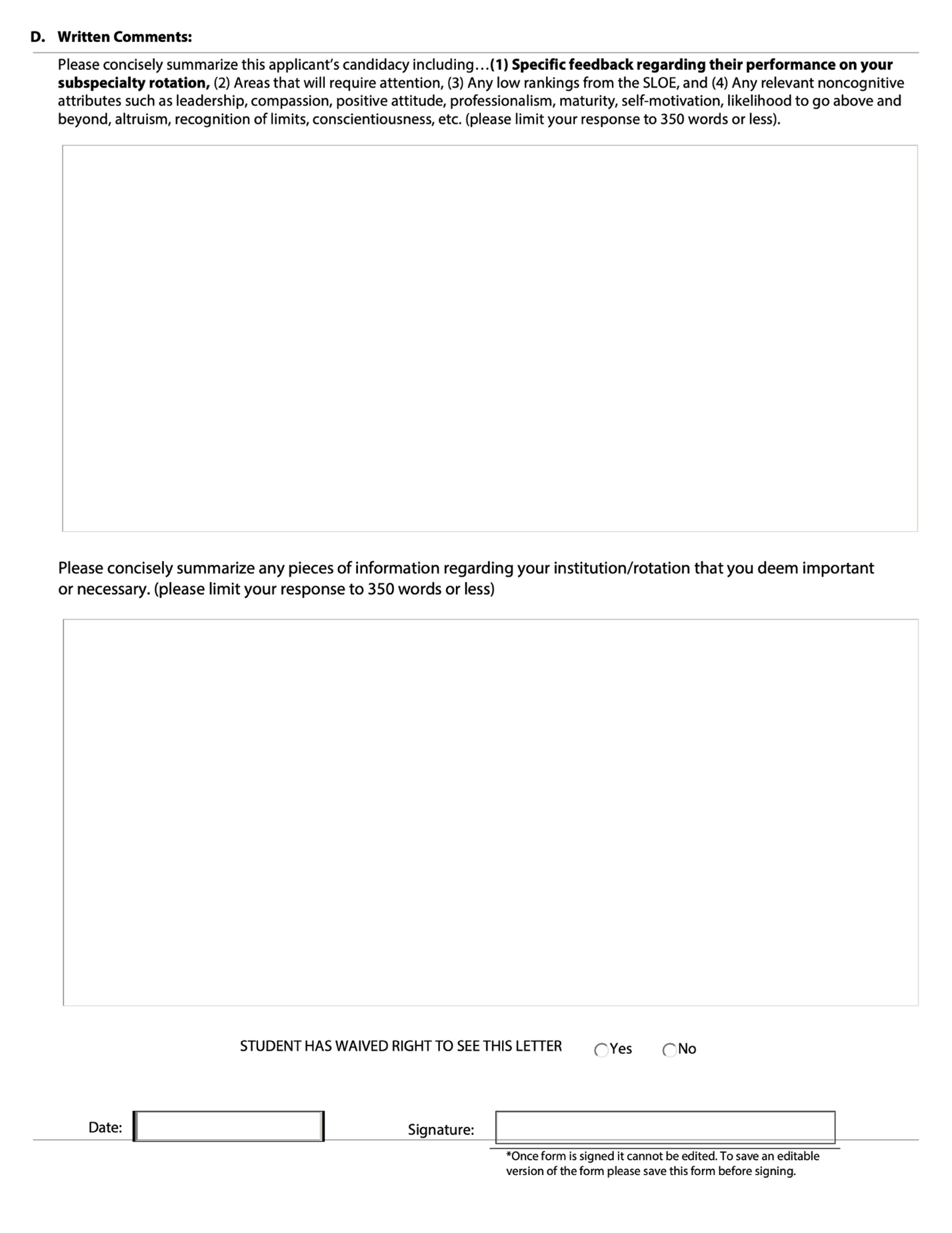

5. 2022 Subspecialty SLOE
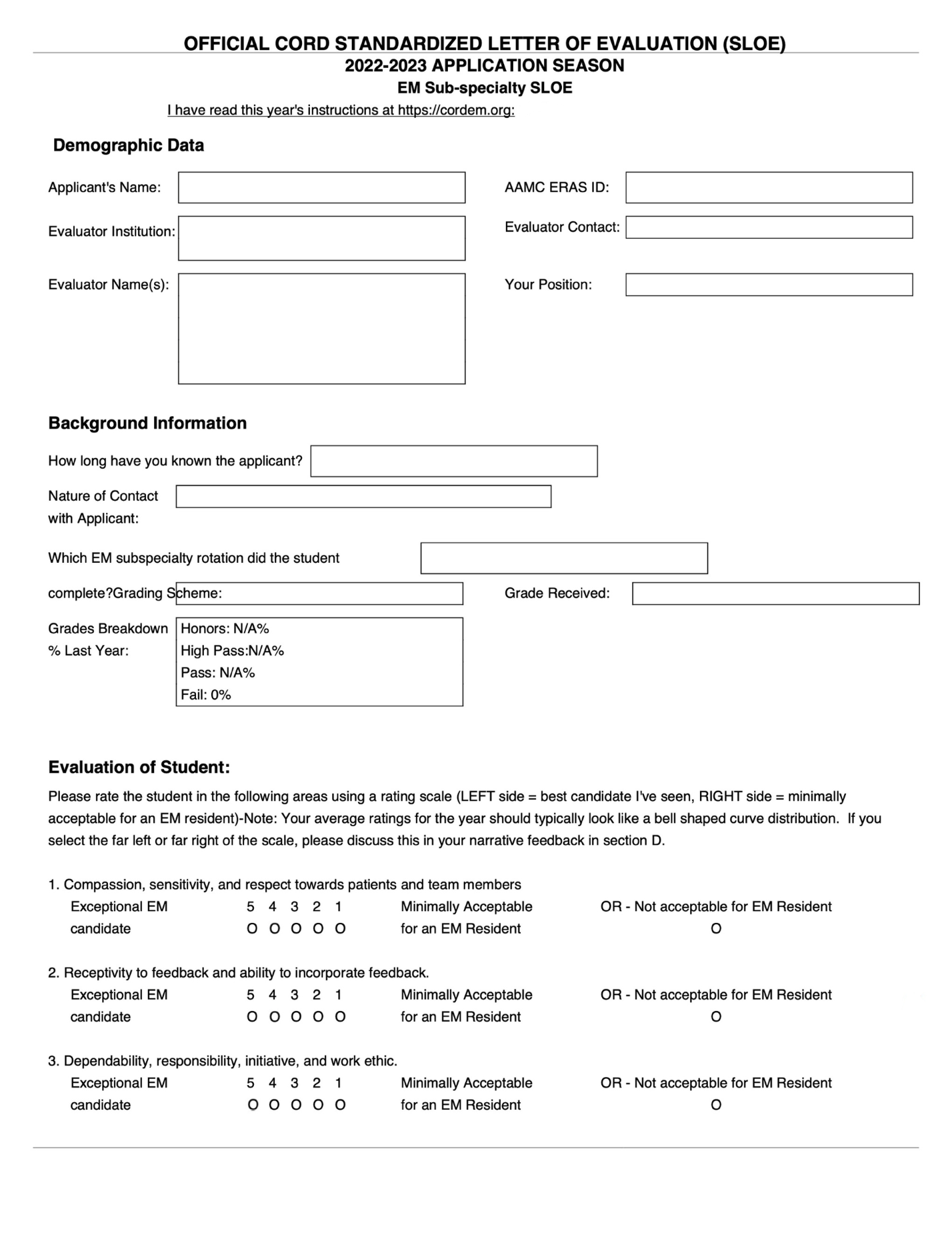

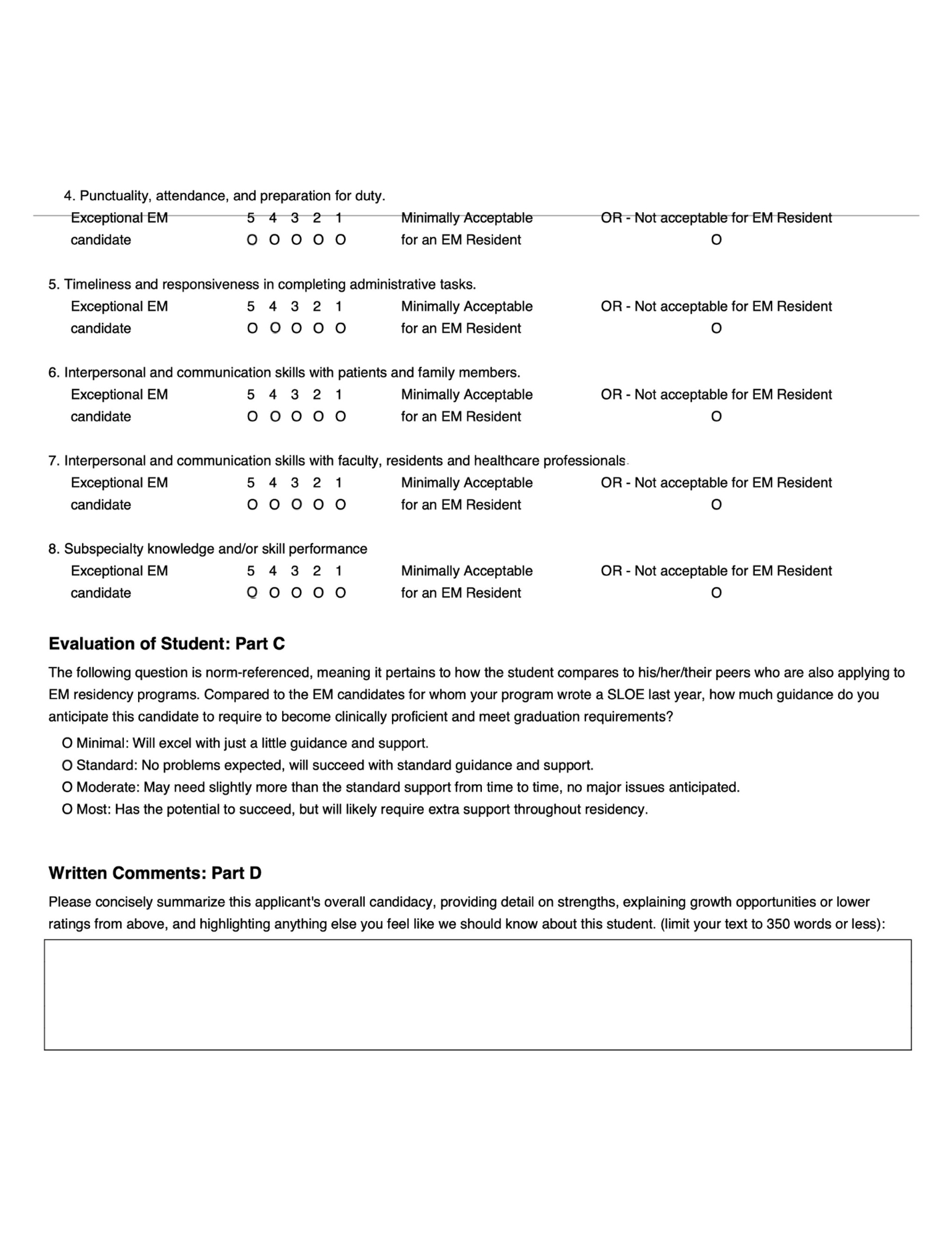

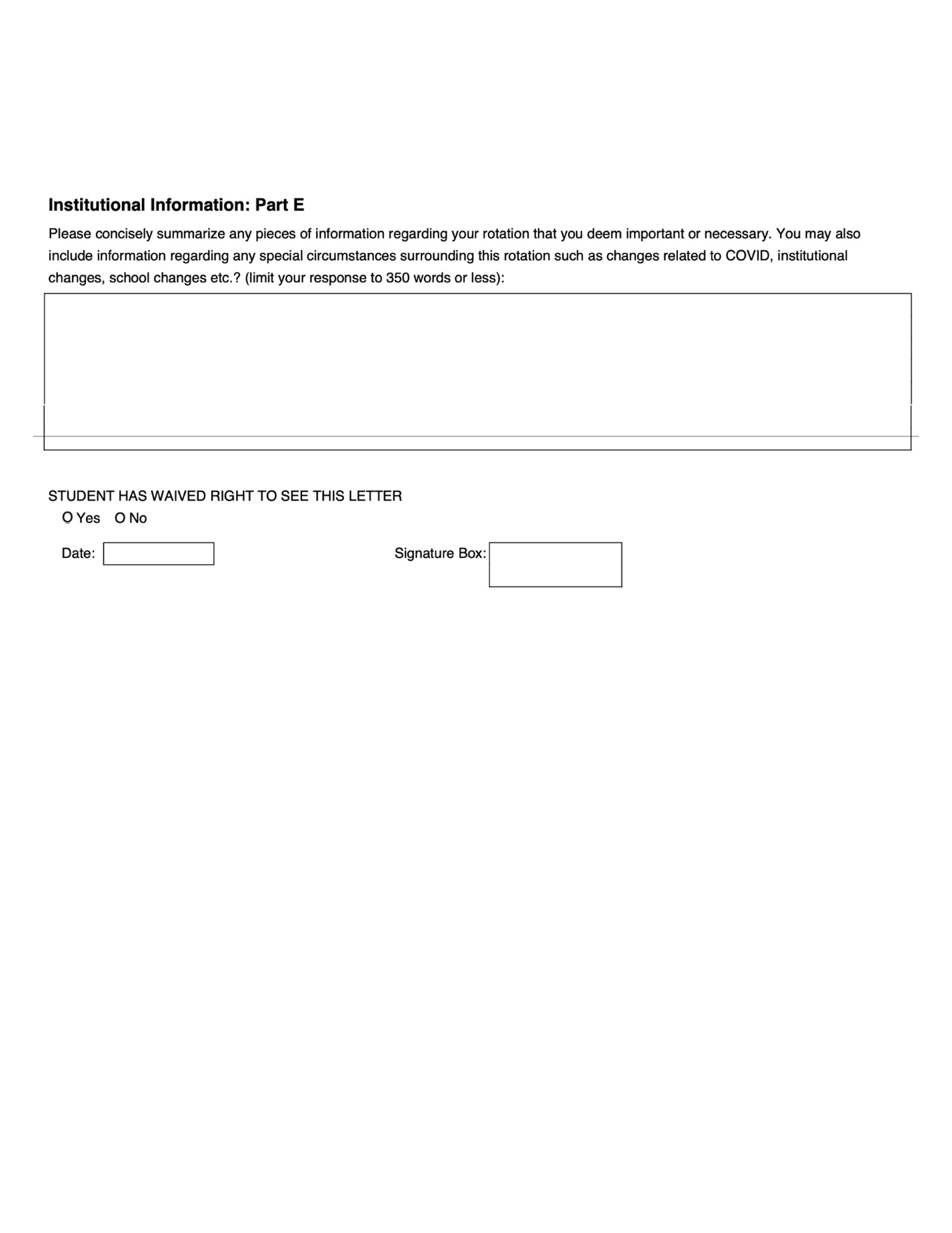

6. 2024 Subspecialty SLOE
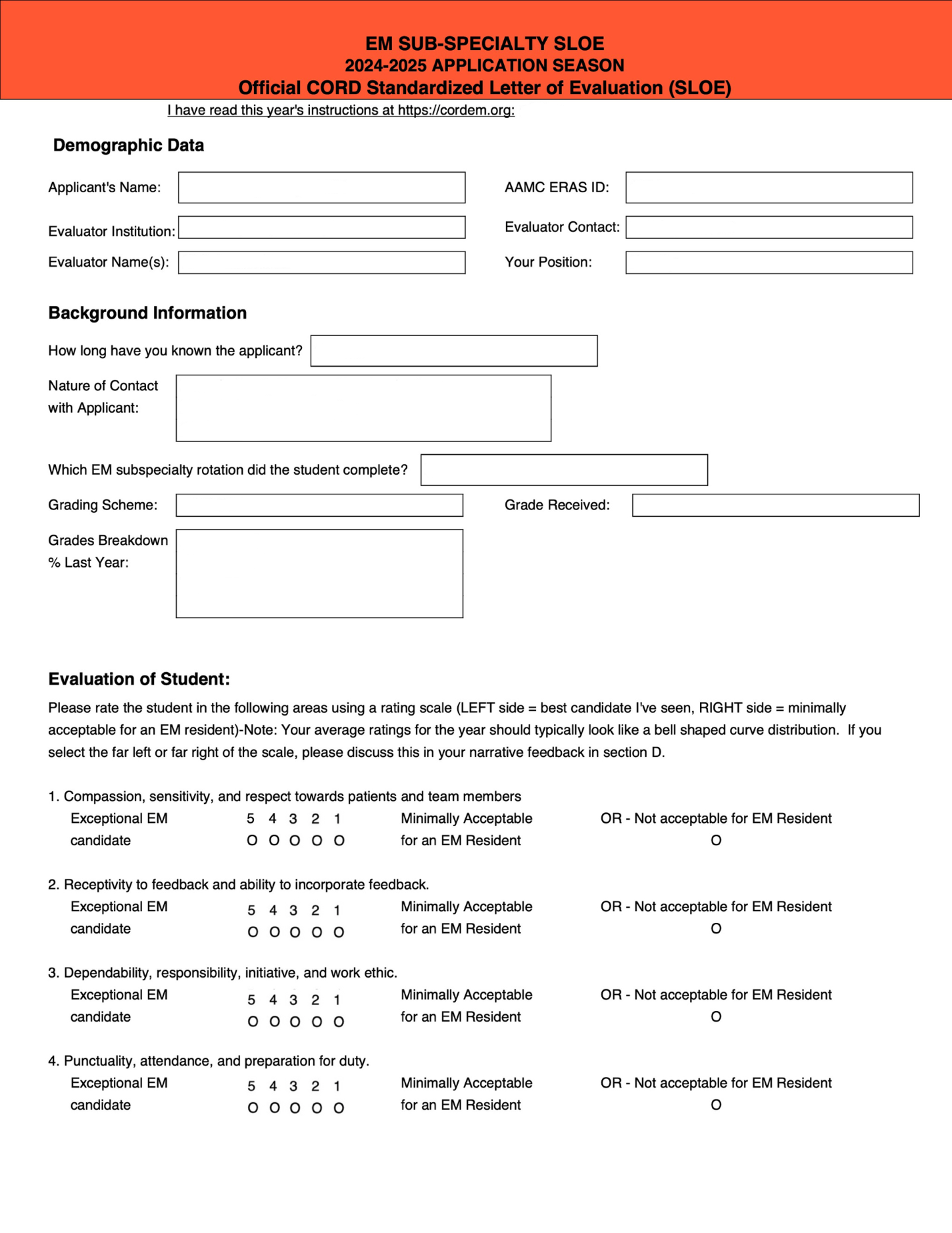

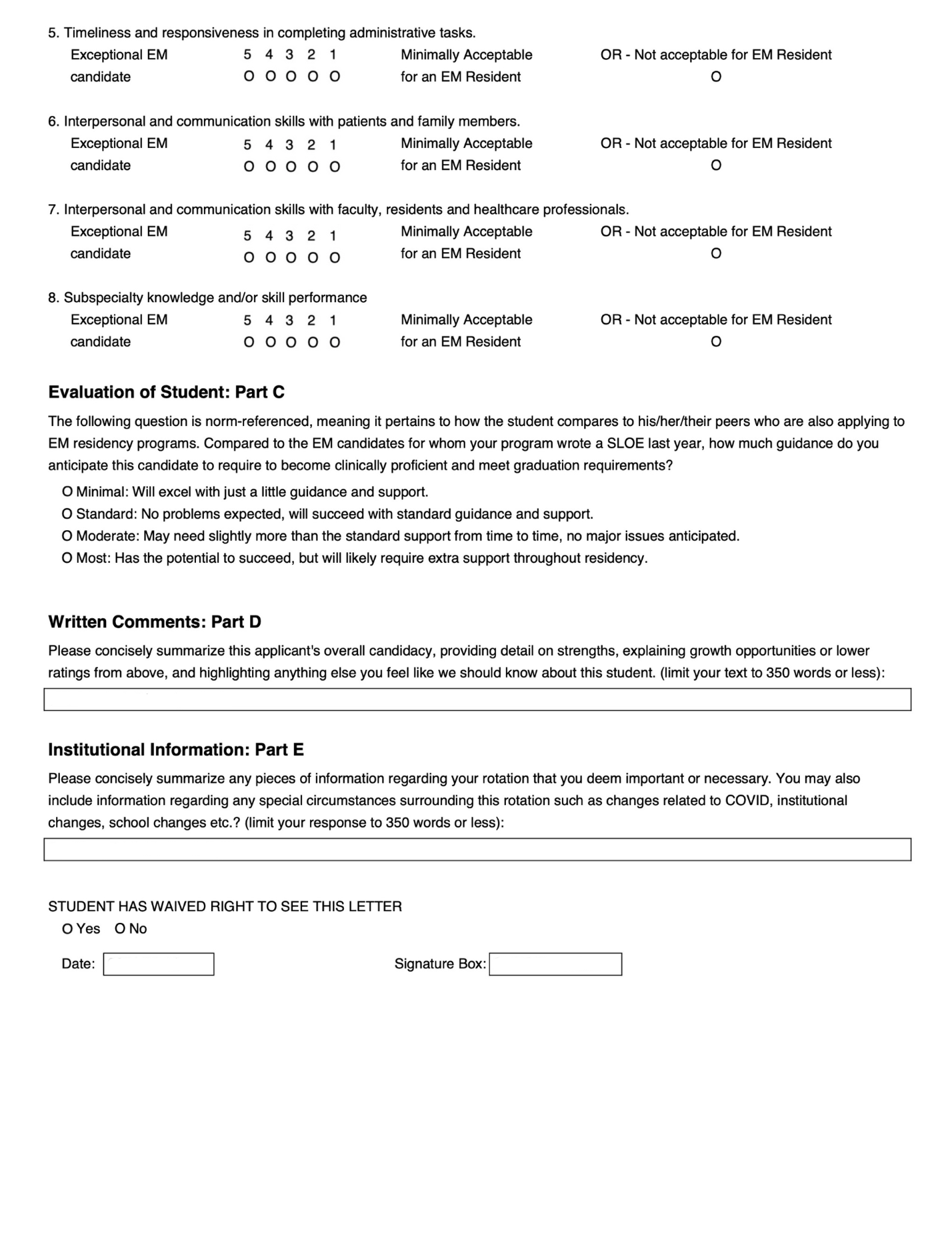

7. 2020 O-SLOE
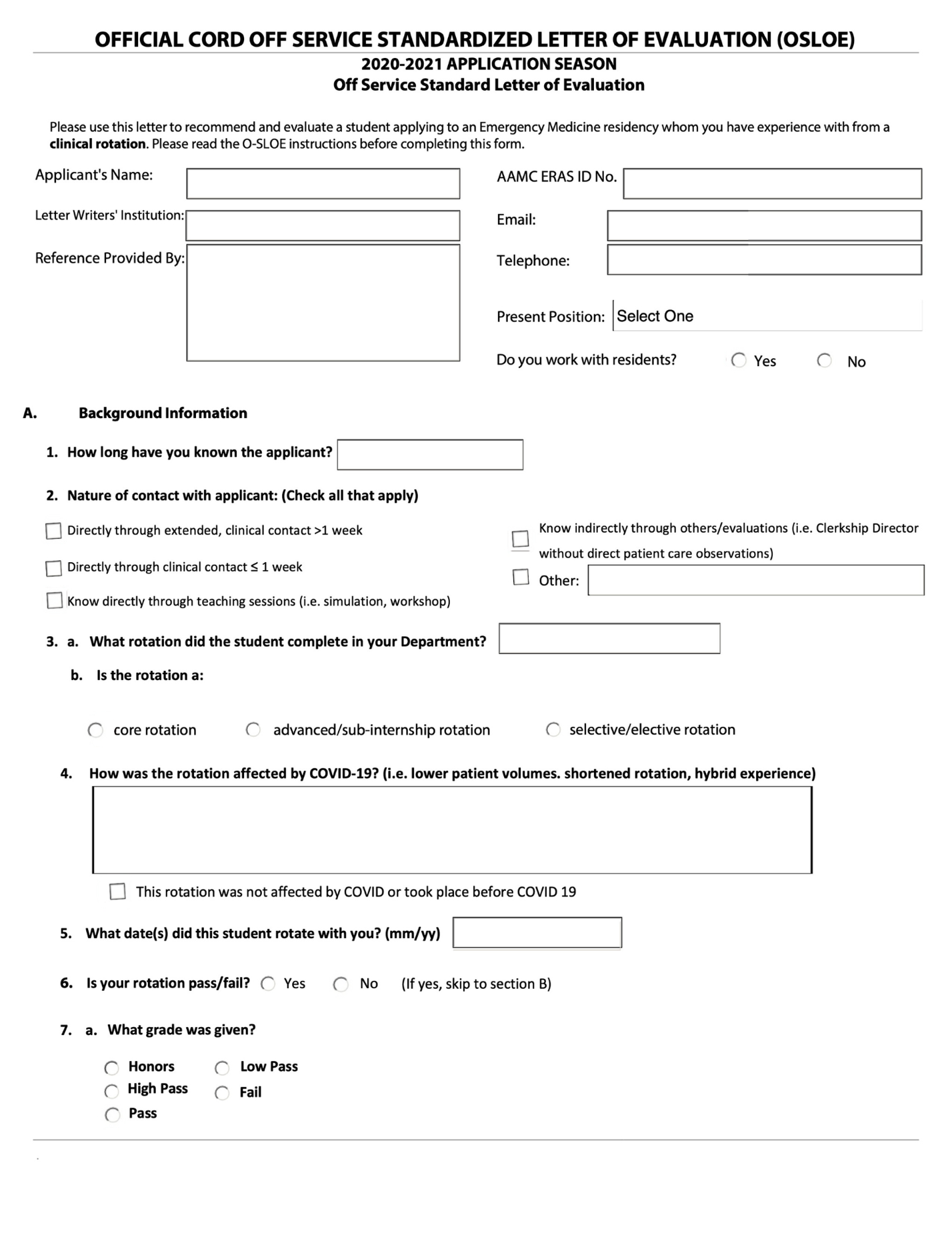

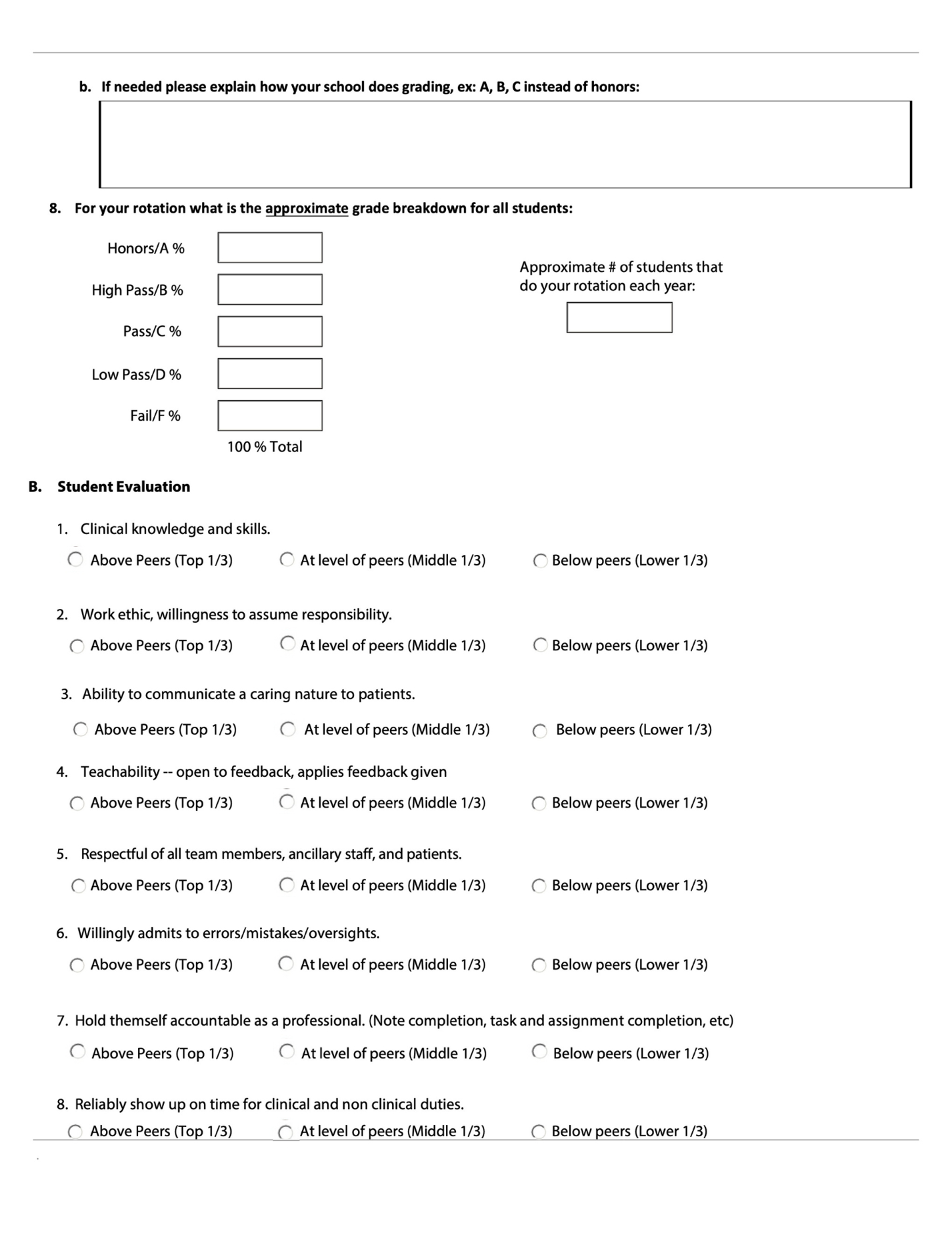

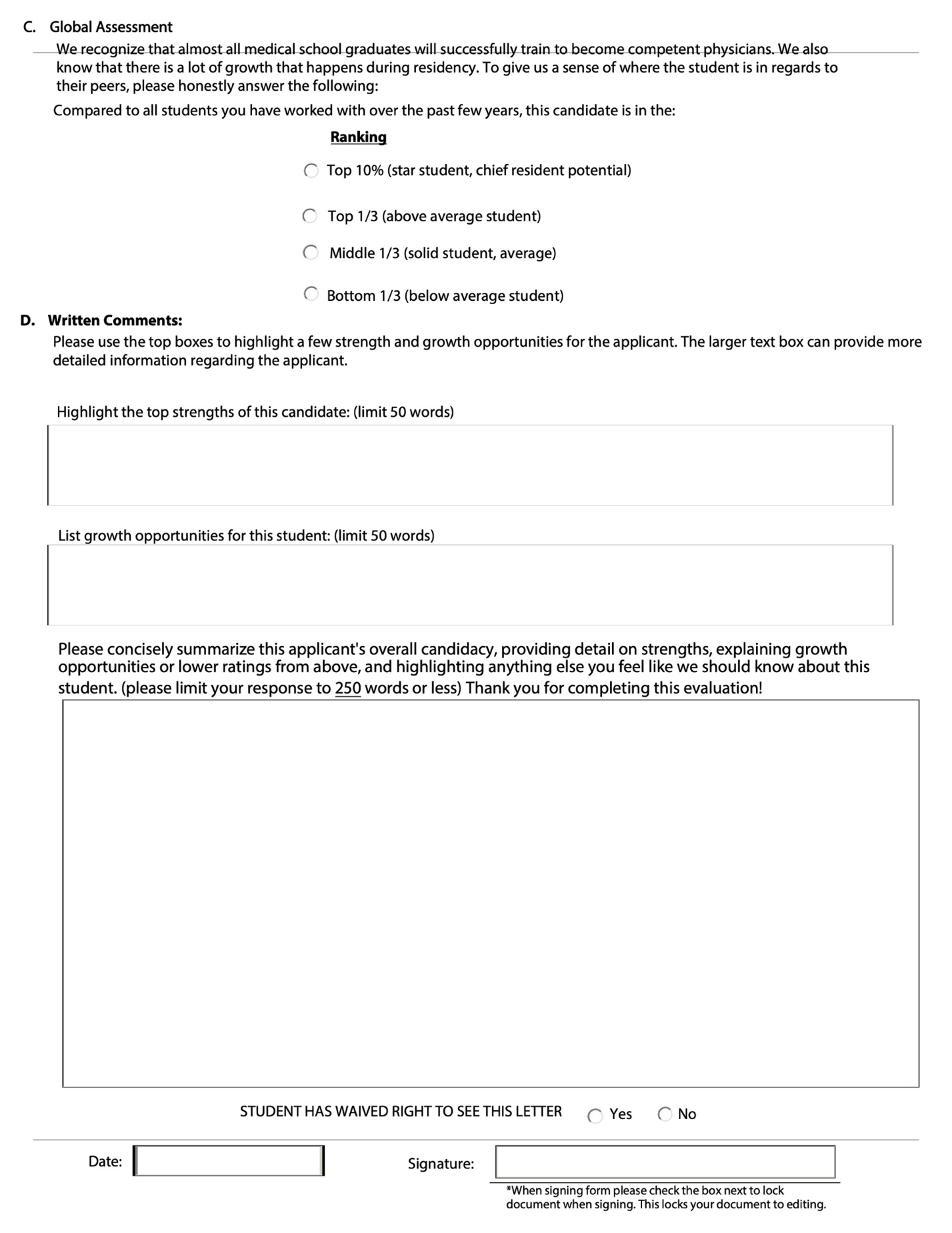

8. 2022 O-SLOE
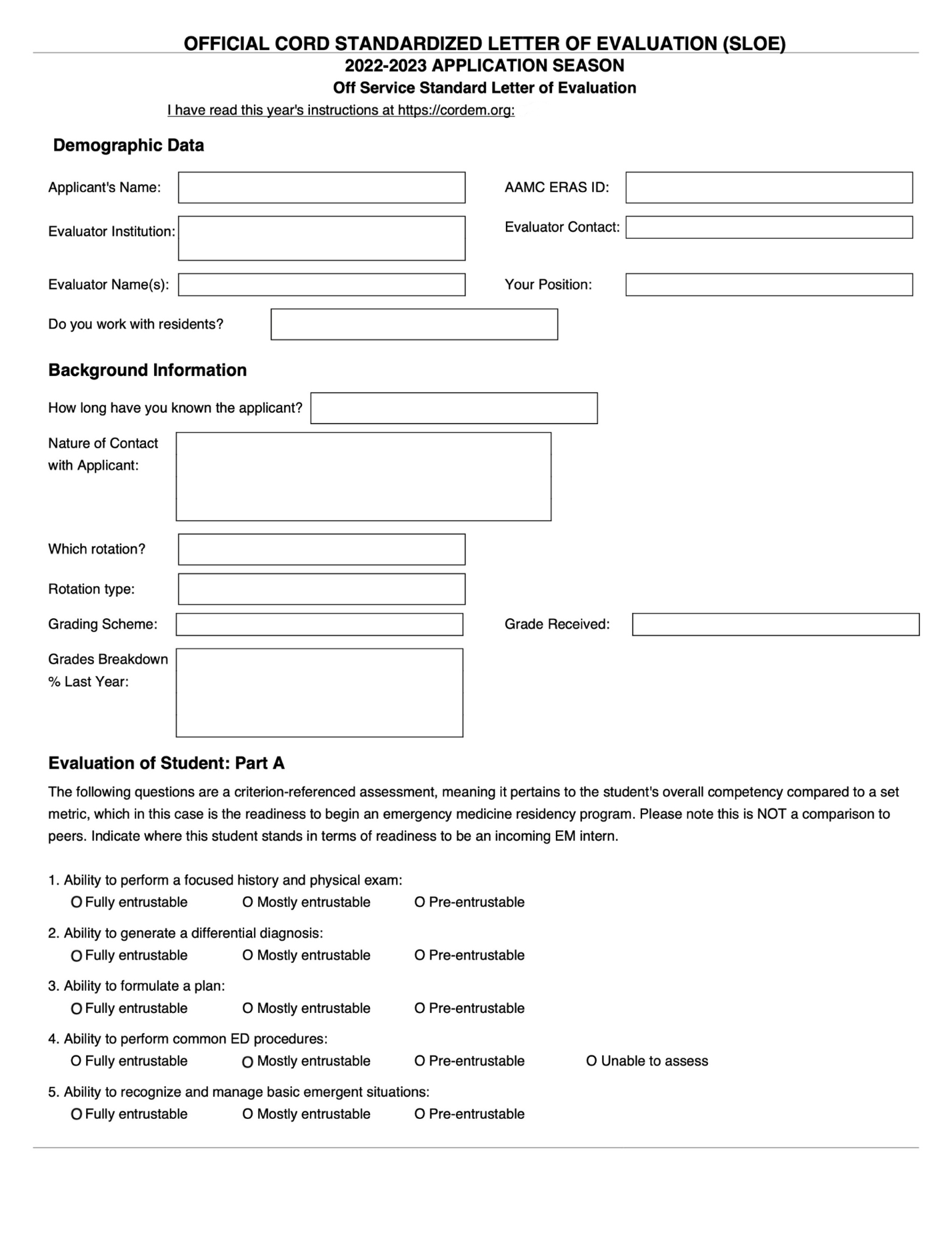

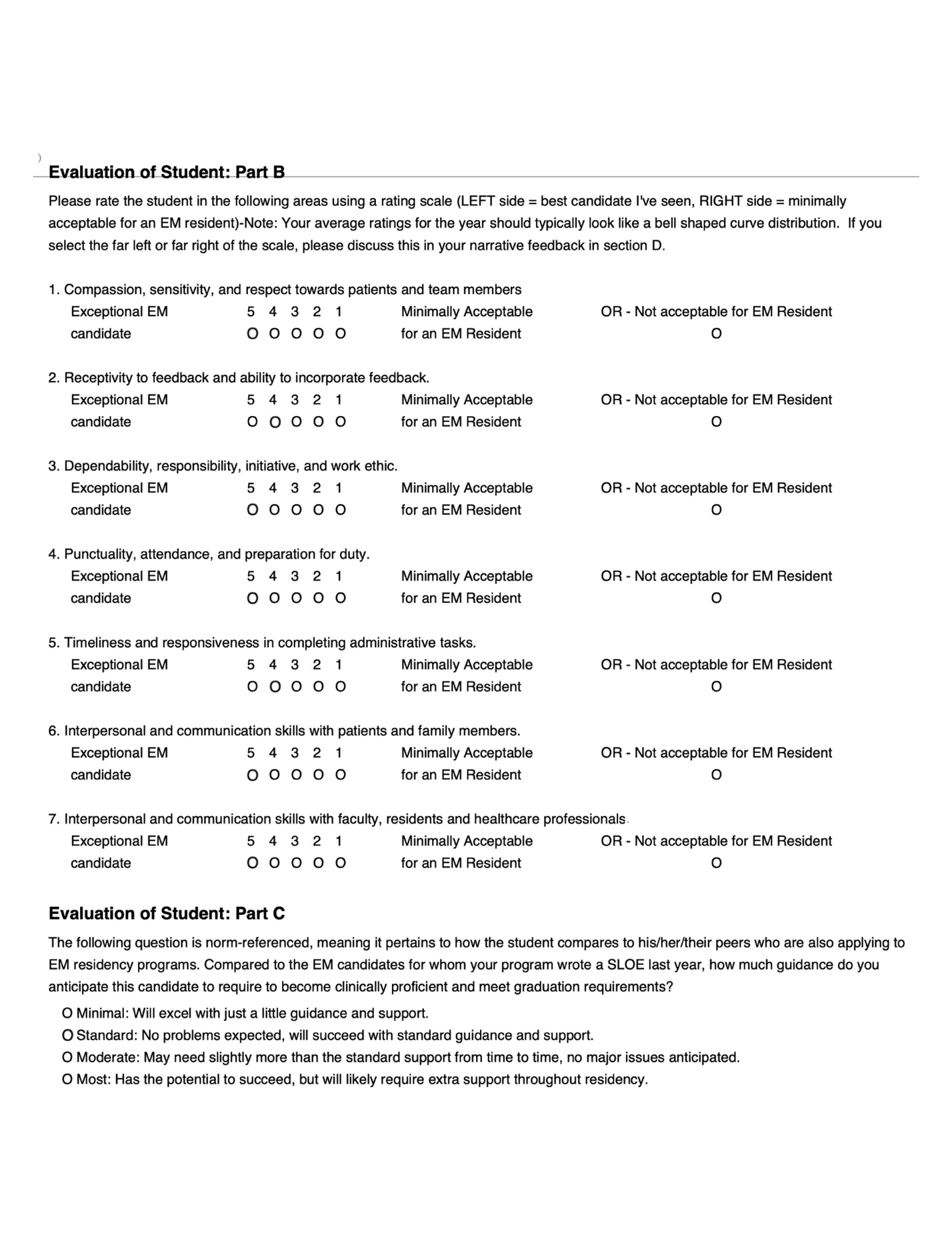

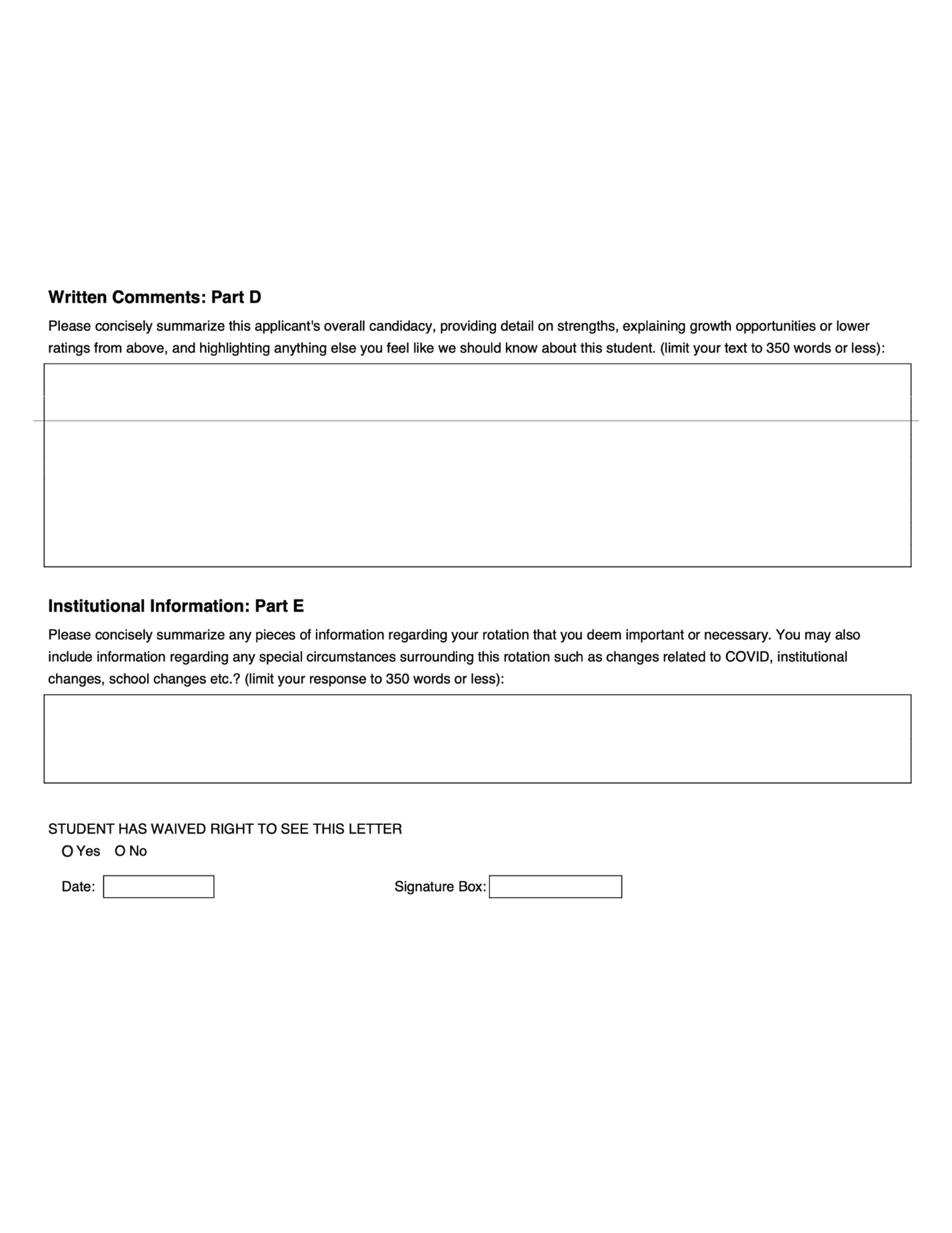

9. 2024 O-SLOE
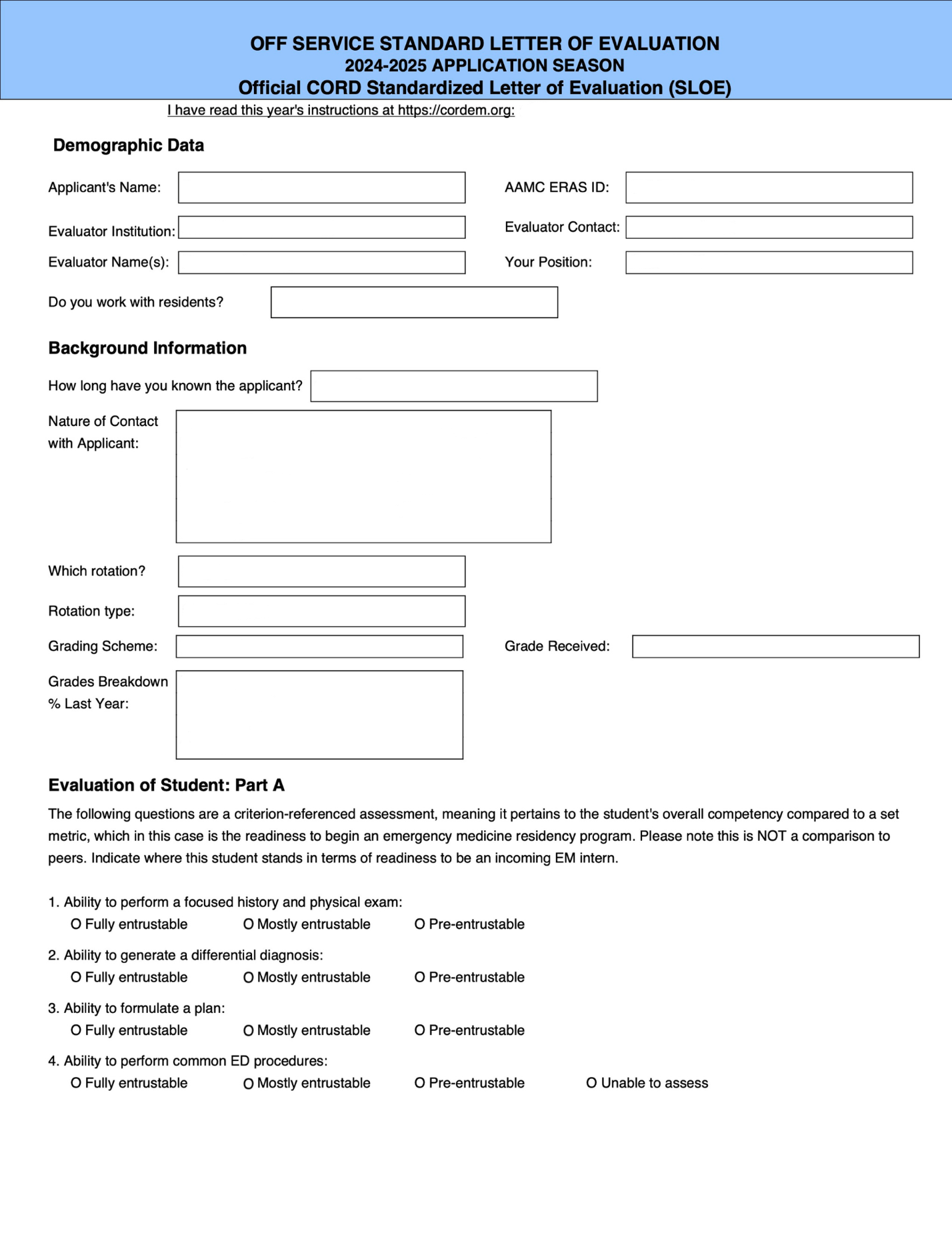

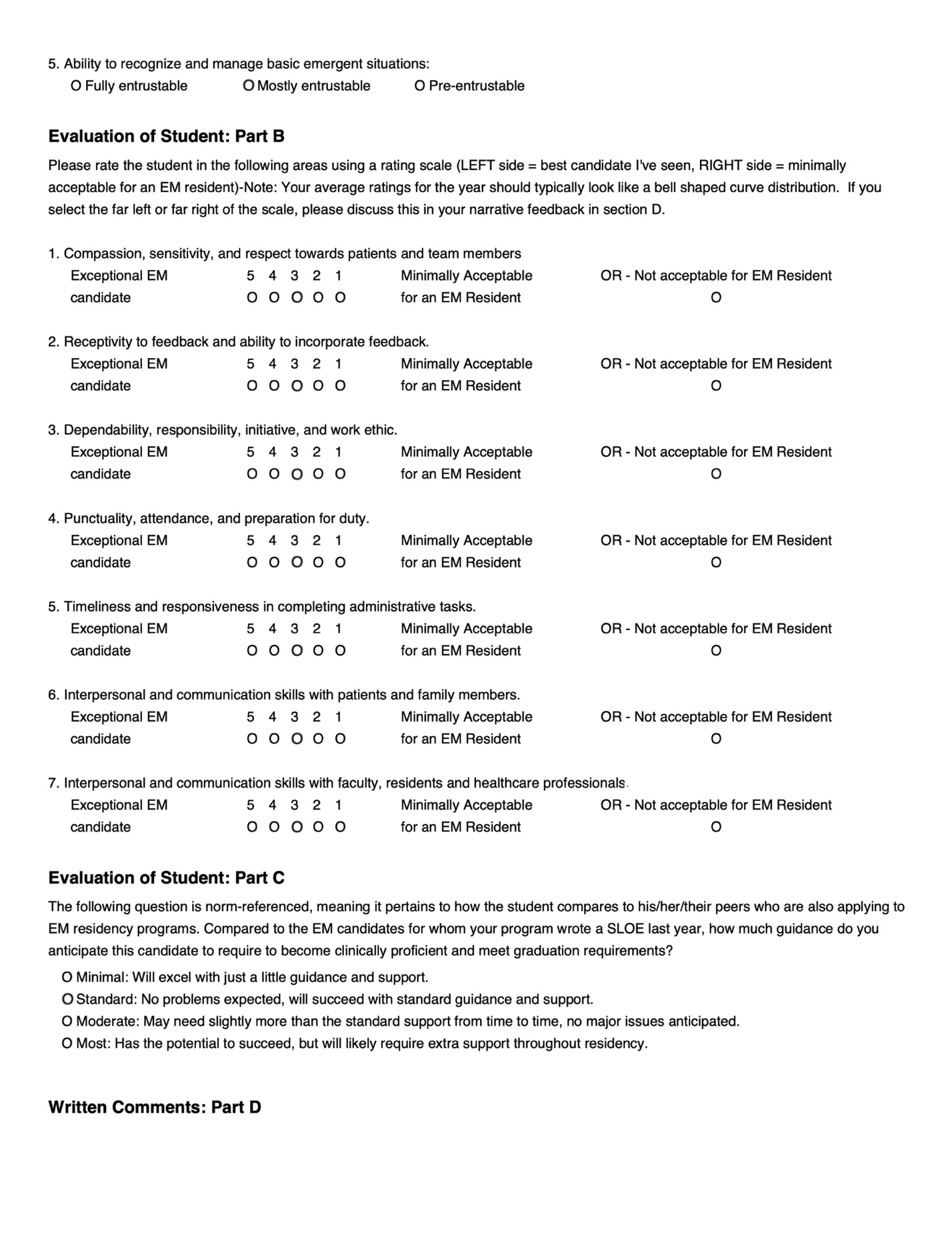

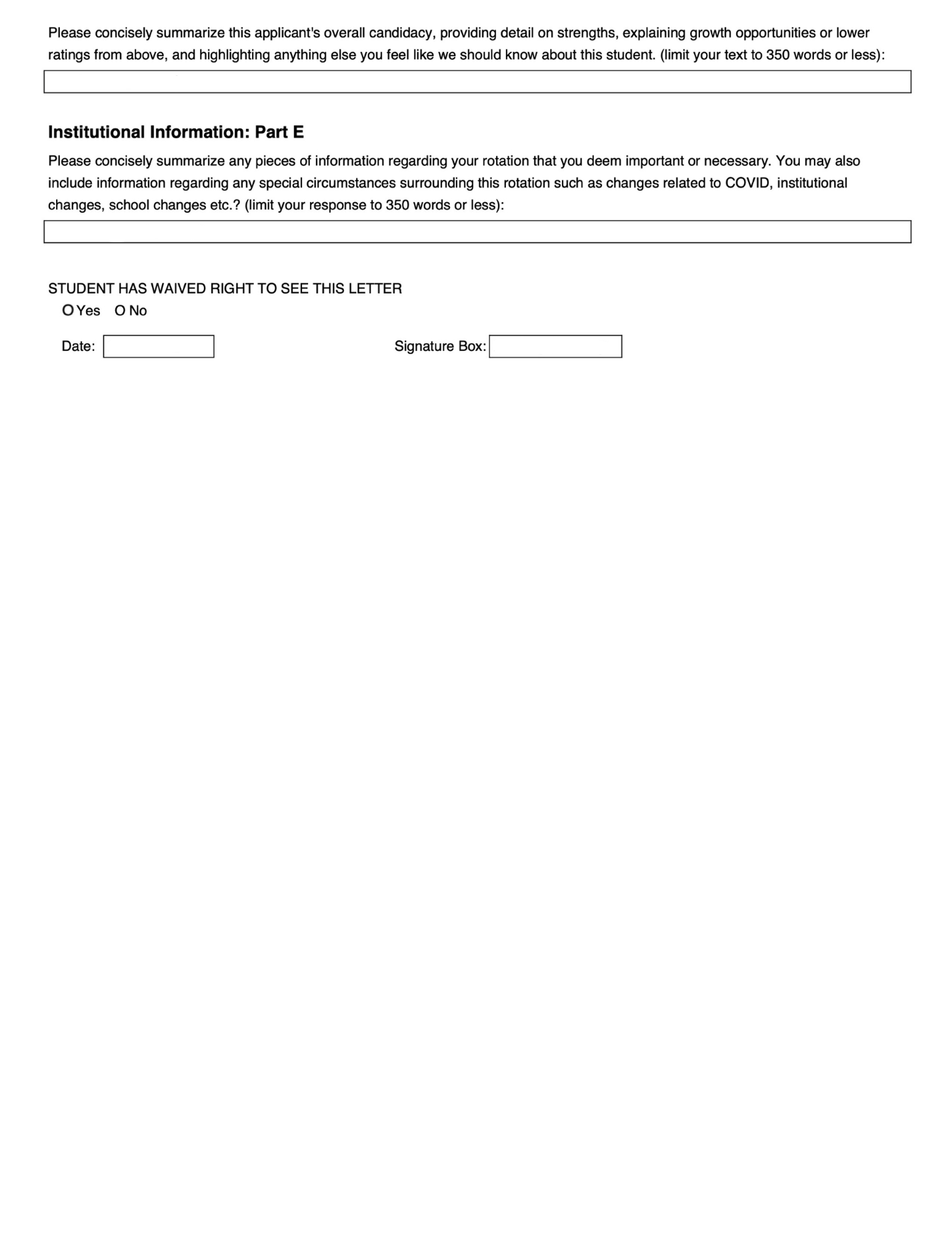


| Version title | Appendix Label |
| --- | --- |
| EM SLOEs | |
| 1995 SLOR | 1A |
| 2012 SLOE | 1B |
| 2016 SLOE | 1C |
| 2020 SLOE | 1D |
| 2022 SLOE | 1E |
| EM SLOE Variants | |
| 2020 SLOE for Non-Academic Emergency Physicians | 2A |
| 2022 SLOE for Non-Academic Emergency Physicians | 2B |
| 2024 SLOE for Non-Academic Emergency Physicians | 2C |
| 2020 Subspecialty SLOE | 2D |
| 2022 Subspecialty SLOE | 2E |
| 2024 Subspecialty SLOE | 2F |
| 2020 O-SLOE | 2G |
| 2022 O-SLOE | 2H |
| 2024 O-SLOE | 2I |
